# Three-dimensional control of alluvial fans by rock uplift in an extensional regime: Aydın Range, Aegean extensional province

**DOI:** 10.1038/s41598-022-19795-0

**Published:** 2022-09-12

**Authors:** Emrah Özpolat, Cengiz Yıldırım, Tolga Görüm, John C. Gosse, Eren Şahiner, M. Akif Sarıkaya, Lewis A. Owen

**Affiliations:** 1 Department of Geology and Environmental Science, University of Pittsburgh, Pittsburgh, PA 15260 USA; 2 Eurasia Institute of Earth Science, Istanbul Technical University, 34469 Istanbul, Turkey; 3 Department of Earth and Environmental Sciences, Dalhousie University, 1355 Oxford Street, Halifax, NS B3H 4R2 Canada; 4 Luminescence Dating Laboratory, Institute of Nuclear Science, Ankara University, 06800 Ankara, Turkey; 5 Department of Marine, Earth, and Atmospheric Science, North Carolina State University, Raleigh, NC 27695 USA

**Keywords:** Geomorphology, Tectonics

## Abstract

Tectonics imparts a first-order control on the overall morphology of alluvial fan systems in extensional settings by influencing sediment flux and accommodation space, while other factors such as climate, catchment lithology, and fault footwall characteristics are secondary. Previous alluvial fan modeling studies have focused on the link between the three-dimensional development of alluvial fans and rock uplift, however, despite the potential influence of tectonics on the overall three-dimensional morphology of alluvial fans, the controlling mechanisms, as well as their relative importance, remain largely unquantified in a natural setting with a targeted source-to-sink approach. Here, we examine 45 alluvial fans and their catchments along the southern mountain front of the Aydın Range, delimited by segmented normal faults in the western Anatolia Extensional Province, to quantify the role of rock uplift. We quantify river incision rates and catchment-wide erosion rates together with a series of topographic analyses across the southern flank of the Aydın Range as a proxy for rock uplift. Our results indicate that the spatial distribution of thicker and steeper alluvial fans fit well with higher rock uplift rates along the strike of the mountain front. In contrast, a lower uplift rate is responsible for prograding alluvial fans with decreasing thickness and gradients. Also, our data shows that alluvial fan thickness compared to other alluvial fan metrics strongly associated with the pattern of the rock uplift. This study demonstrates a field-based, quantitative linkage between three-dimensional alluvial fan morphology and rock uplift which has significant implications for improving alluvial fan models and understanding how alluvial fans respond to tectonics in extensional regions.

## Introduction

Alluvial fans are the most dominant depositional landforms developed along the mountain fronts delimited by normal faults in extensional settings. Their morphology and sedimentological features are controlled by tectonics^1–5^, climate^6,7^, lithology^8,9^, and drainage basin characteristics^10–12^. Alluvial fans have been heralded as sensitive indicators of tectonic in extensional settings and alluvial fan deposits-morphology as indicators of movement on basin-bounding faults paleogeography. For these reasons, there has been great interest in exploring the relations between the morphological properties of alluvial fans and tectonics.

Previous analog and computational models have revealed a positive correlation between alluvial fan thickness and the tectonic uplift of their contributing catchments observing the three-dimensional development of alluvial fans^13,14^. In contrast to earlier modeling studies, field-based alluvial fan morphometric studies mostly have focused on relations between planimetric features of alluvial fans and their catchments, including e.g., the ratio between the fan area and the catchment area^15–17^, and the ratio between the alluvial fan slope and the average slope in the catchment^4^. Most alluvial fan morphometric studies presumed alluvial fan areas are a proxy for sediment yield from its catchment^18–20^. They would expect a good correlation between the catchment area and the fan volume, which is, however, usually not known nor computed. The alluvial fan volume is, therefore, represented by the planimetric fan area that is applied as a proxy for sediment yield and does not consider the thickness of the alluvial fan which may differ, depending on the structure of the mountain front and the accommodation space^21,22^.

Nevertheless, using the only 2D planimetric area of alluvial fans may lead to inaccurate assessments of the sediment-flux and ranking of the controls of alluvial fan development because their volume also depends on their thicknesses. For instance, variations in alluvial fan thickness may result in different volumes for alluvial fans with the same planimetric area^22^. Given the challenges in obtaining reliable volumetric data on a sufficient number of alluvial fans for a given extensional setting, the relationship between the three-dimensional morphology of an alluvial fan system and the history of rock uplift of the source footwall block has yet to be rigorously quantified in natural landscapes. Therefore, a three-dimensional investigation of alluvial fans may provide new insights into a better understanding of interaction, especially between tectonic and sedimentary flux in natural landscapes. Additionally, a controlled region with numerous alluvial fans, volumetric data, local uplift data, and low variability in non-tectonic factors can provide the natural laboratory needed to clarify the gap between modeling studies and field-based studies.

The Aydın Range in western Anatolia Extensional Province provides a very suitable geomorphic setting to examine the relationship between alluvial fan development and tectonics, mainly because of the numerous alluvial fans in this region. The alluvial fans are aligned along a segmented normal fault^23^, the catchments have not been glaciated over the duration of alluvial fan deposition^24^, the climate, and vegetation, within each catchment-alluvial fan system are very similar, and there is a mixture of mostly metamorphic and plutonic lithologies with some sedimentary rocks that are mapped^25–27^. Furthermore, the area offers a wide range of river incision and catchment-wide erosion rates^24,28^, as shown here. We interpret these as a function of differential rock uplift between the footwall catchments and hanging wall alluvial fan systems. Importantly, there is no apparent reason, e.g., along-strike thermal anomalies or variances in lithospheric elastic thickness, to expect that the ratio of isostatic and tectonic contributions to the rock uplift varies along the range front, so it appears that different combinations explain the desired variation in the along-strike rates of uplift from a high angle normal faulting. Additionally, the region is well suited for a topographic analysis, with sparse vegetation and minimal human disturbance, to examine the influence of allogenic and autogenic driving factors on the three-dimensional development of alluvial fans. Thus, the principal objective is to investigate the relationship between the three-dimensional morphology of alluvial fans and relative rock uplift patterns in a tectonically active region where differential uplift rates span a sufficiently wide range.

## Study area

**Figure 1:**
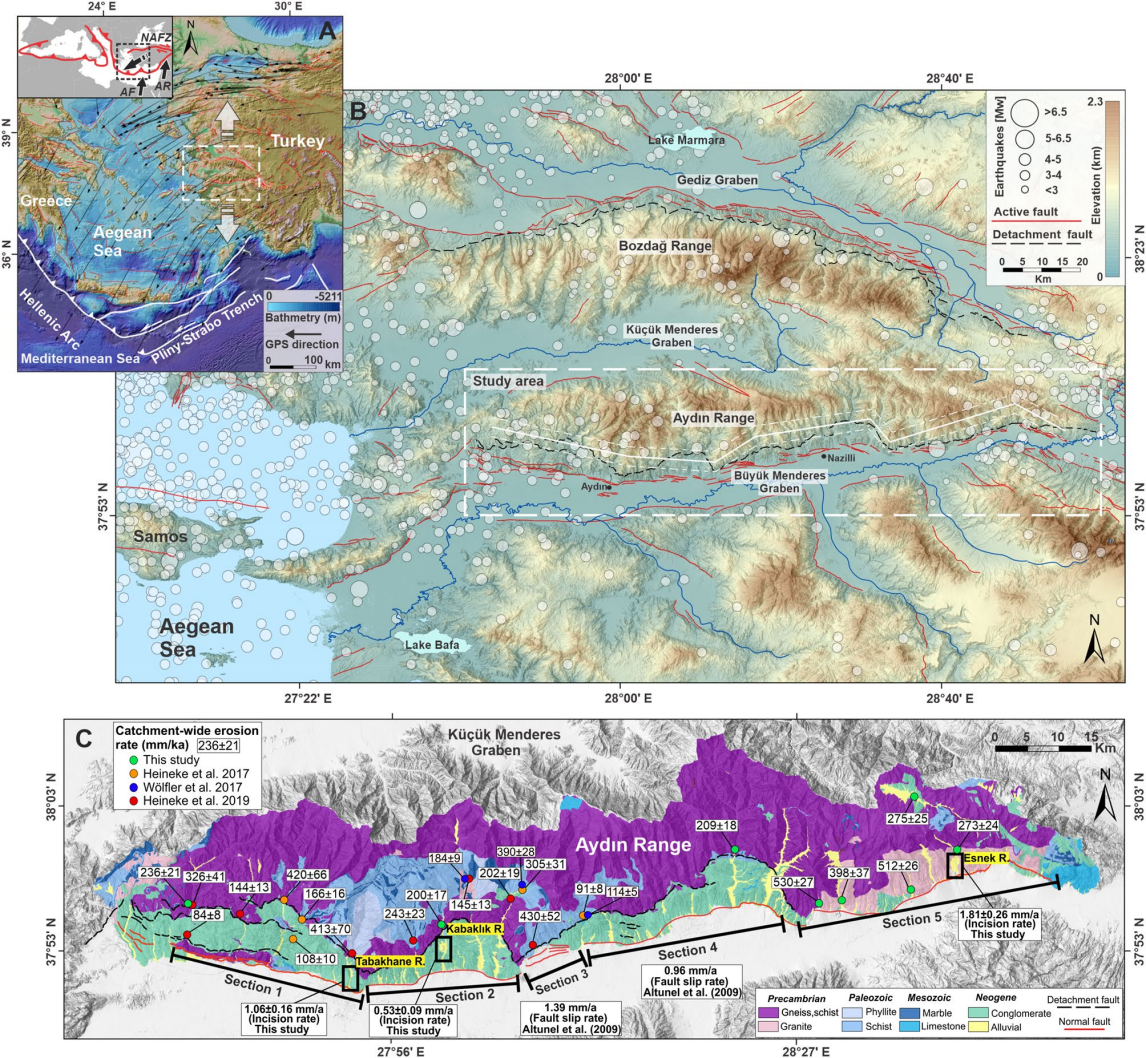
(**A**) Tectonic setting of the Aegean Region. GPS vectors are from Reilinger et al.^30^. White arrows show extensional movement. *NAFZ* North Anatolian Fault Zone, *AF* African Plate, *AP* Arabian Plate. (**B**) Topographic setting of western Anatolia Extensional Province with active faults^23^ and earthquake epicenters from the USGS. The white swath profile shows the relief in Fig. 3F). (**C**) Lithological map of the study area with catchment-wide erosion and incision rates. (Lithological map was compiled from previous studies^25–27,44^ and General Directorate of Mineral Research and Exploration (MTA)^45^. Black rectangle boxes indicate river terrace locations where river incision rates were obtained. White boxes indicate incision and fault slip rates associated with each section.

The western Anatolia Extensional Province in the Aegean region is one of the most rapidly extending regions on Earth at a rate of ∼ 20 mm/year in the N–S direction, owing to the subduction of the African plate along the Hellenic trench^29–31^ (Fig. 1A). Quaternary extension led to the formation of E–W oriented horst and grabens delimited by normal faults, which is part of the Menderes Massif Metamorphic Core Complex^32,33^. The Central Menderes Massif is located at the center of this rapid N–S extension and the massif is subdivided to three-part into the northern Bozdağ and the southern Aydın Range which are separated by the Küçük Menderes Graben and bound by the Gediz and Büyük Menderes grabens in the north and south, respectively. The boundary between the Büyük Menderes Graben (BMG) and Aydın Range is defined by the low angle Büyük Menderes Detachment Fault and its high angle synthetic faults^34^ (Fig. 1B).

Aydın Range has experienced an uplift as a result of initial extension causing low-angle detachment faults along its southern flank since the early Miocene^32–36^. Geophysical and drilling studies^37,38^ have indicated a normal faulting structure, resulting in stepwise graben formation, which is also characteristic of the Büyük Menderes Graben. These E–W striking normal faults cut across nearly 100-m-thick cataclastic, which were formed at the base of Büyük Menderes Detachment Fault (BMDF), and non-metamorphic Neogene sediments on the crystalline basement^39,40^. In contrast to BMDF, the age of the normal faults in the latter stage activating following the BMDF is not constrained exactly and their timing is controversial^41^. Altunel et al.^42^ estimated slip rates of the high-angle normal faults based on trench studies and archeo-seismological data. They showed that the Sultanhisar fault segment (“Section 3” in our study) has a higher slip rate in contrast to Nazilli Segment (“Section 4” in our study). Additionally, Barbot and Weiss^43^ produced a kinematic model of surface deformation in Aydin Range using Median Interannual Difference Adjusted for Skewness and Global Navigation Satellite System data acquired between 1995 and 2020. Their results indicated that the eastern part of the Aydın Range (“Section 5” in our study) has higher slip rates compared to the western (“Section 1” in our study) and central part (“Section 4” in our study), relatively. Also, it is well documented that the southern flank of the Aydin Range is characterized by fault escarpments, raised river terraces, triangular facets, and a high-relief landscape that has been incised by rivers draining to the BMG^25,26,44^. These previous studies suggest that Aydın Range is still undergoing differential rock uplift most evidently due to the high-angle segmented normal faulting along its southern flank. The sediments trapped from erosion in the uplifted Aydın Range (footwall block) have formed alluvial fans in the hanging wall along the northern margin of the BMG^26,44^. We examine 45 alluvial fans and their catchments that drain to the BMG from the southern flank of the Aydın Range (Fig. 1C).

## Approach

We present a quantitative analysis of the controls on the three-dimensional morphology of alluvial fans. Although Aydın Range in western Anatolia Extensional Province is one of the most-studied horsts in western Turkey in terms of the active tectonic, absolute data on rock uplift is still lacking in some parts of the range. Thus, we subdivide the analysis into two parts: (1) constraining the rock uplift and erosion pattern across the southern flank of the Aydın Range, and (2) computing the alluvial fan volumetric–planimetric parameters. Finally, we combine all data from alluvial fans and source footwall block to investigate the role of the rock uplift on the 3D morphology of alluvial fans.

The three river terrace staircases investigated in this study are located on footwall block controlled by high-angle normal faults along the southern flank of the Aydın Range. The Büyük Menderes Graben forming the hanging-wall block is a local base level for rivers flowing south from the Aydın Block (Fig. 1C). So, these river terraces can provide significant insights into Spatio-temporal variations in rock uplift. Tabakhane and Kabaklık river terrace staircases are located on Neogene terrestrial sediments which deposited following the development of the Büyük Menderes Detachment Fault^35^. These Neogene-aged paleo-fan deposits called Tmolosschutt by Philippson^46^ and Erinç^47^ were well constrained by mammal fossils and thermochronological approaches^35,48–50^. Also, it is well documented that these Neogene deposits, on which the river terraces are deformed by the north by high-angle normal faults^25,44^ in the latter stage. Therefore, Tabakhane and Kabaklık river terraces developed on Neogene-aged lithology were considered bedrock river terraces and used for tectonic inferences (Figs. S2, S3). In Esnek River terrace staircases, we observed a strath terrace surface in T1 (Fig. S4). Previous studies^51,52^ showed that strath terrace surfaces are ideal geomorphological markers for the rock uplift rate. We used the river incision rates from the strath terrace surface in Esnek River as a proxy for rock uplift patterns. River incision rates derived from these river terraces in footwall block controlled by normal faults provide a proxy for the rock uplift rate in the Aydın Range. We also analyzed the channel steepness index (*k*_*sn*_), distribution of tectonogenic^53^ slope-break knickpoints, and the relief for interpretation of the uplift along the southern flank of the Aydın range. Long-term river incision rates, normal fault slip rates from earlier studies, and a series of topographic analyses provide significant insight into spatial–temporal variations in rock uplift of the Aydın Range.

To understand the spatial variations in erosion along the southern flank of the Aydın Range, we performed eight ^10^Be-derived catchment-wide erosion rates. These new erosion rates were integrated with previously published 13 catchment-wide erosion rates^24,28,83^ in the study area. To evaluate the possible role of lithology on erosion, lithological units for each catchment were reclassified into igneous, metamorphic, and sedimentary rocks. TRMM data (0.25° × 0.25°) were used to assess the possible role of the variations in precipitation on erosion.

**Figure 2:**
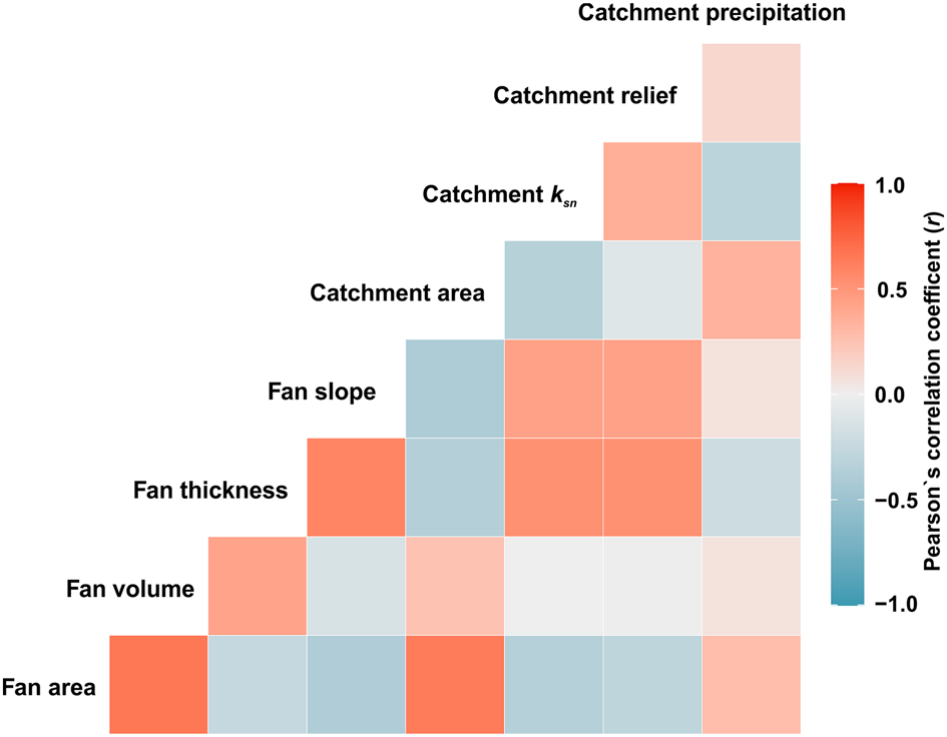
Pearson’s correlation coefficient (*r*) of the eight alluvial fan-catchment variables.

The visible conical alluvial fan volumes enclosed between the base surface and the present-day alluvial fan topography, thickness, area, and gradient were calculated based on TanDEM-X with a 12.5 m cell size. To compute the volume, we assumed a vertical abutment against the mountain-front from extending down. This assumption results in a small overestimate since the mountain-front escarpment dips mostly 45°–72° south. The base level of alluvial fans was specified as an elevation of the alluvial fan toe for each alluvial fan and we assumed a flat base level since fan subsurface data is lacking. This assumption results in a small underestimate of the alluvial fan volume that tends to be larger than the abutment overestimate. Finally, the southern mountain-front of the Aydın Range was divided into five main sections (S1 through S5) based on three-dimensional features of alluvial fans and tectonic and erosional characteristics of footwall block (see the methodology section and supplementary material) (Fig. 2).

## Results

### OSL ages and river incision rates

**Figure 3:**
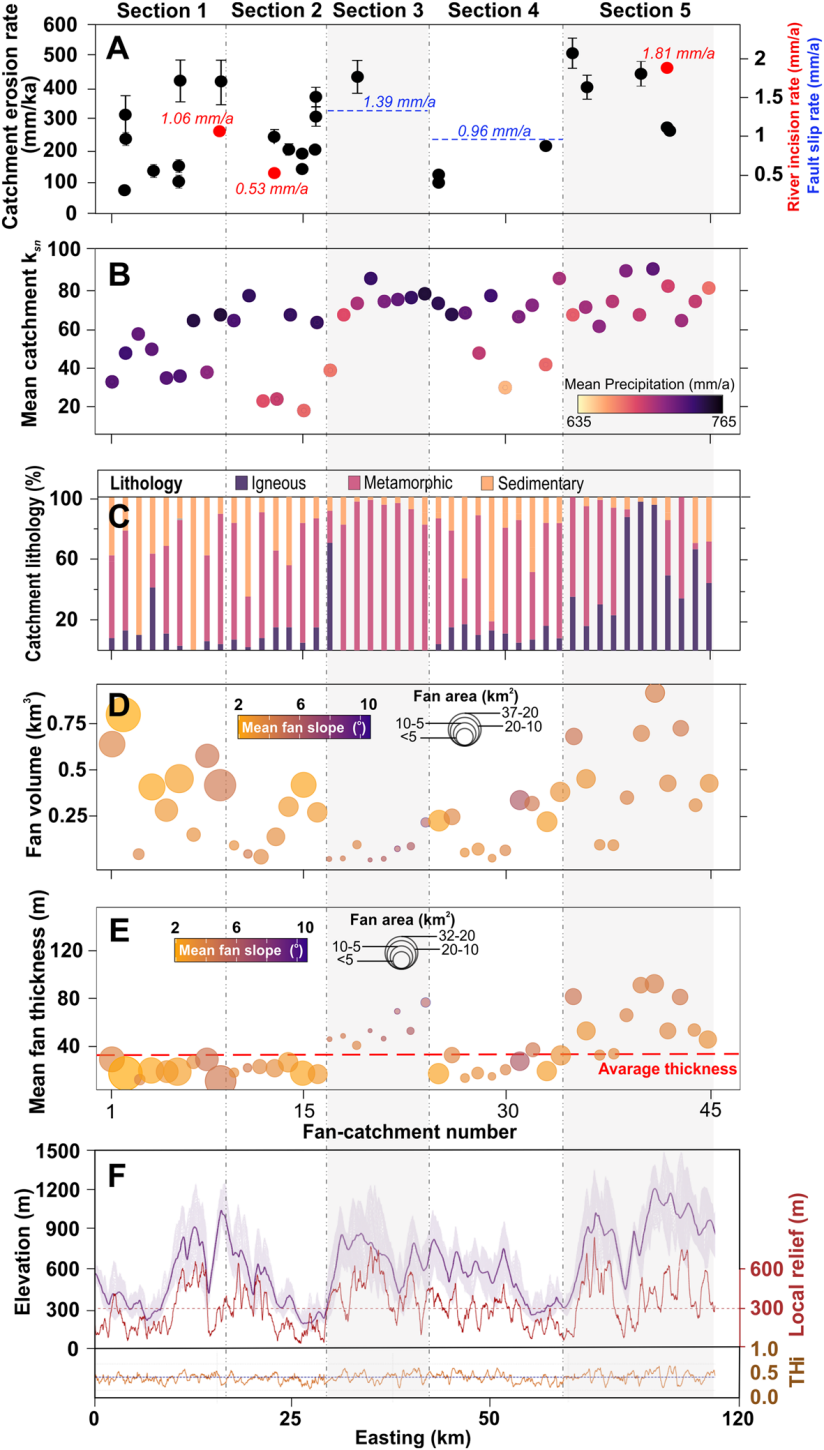
(**A**) The river incision (red dots), catchment-wide erosion rates (black dots), and fault slip rates (blue dots). (**B**) The mean catchment *k*_*sn*_ and mean annual precipitation. (**C**) Relative cumulative proportions of the catchment lithologies. (**D**) Estimated alluvial fan volume, area, and slope. (**E**) Alluvial fan thickness, area, and slope. The dashed red line shows the mean alluvial fan thickness. (**F**) Local relief within a 1-km-radius circular moving window. *THi* transverse hypsometric integral. See Fig. 1B for the locality of the swath profile. The dashed black line shows sections and the grey shaded color marks a higher rock uplift rate relatively (see the text for details).

Our eight OSL ages are within the late Quaternary ranging from 69 ± 10 to 6 ± 2 years (Table S2). The OSL samples collected as close to the river terrace surface as possible represent the abandonment age of terrace surfaces. OSL samples from the terrace staircases of Tabakhane River in “Section 1” yielded ages between 69 ± 10 ka (T1) and 47 ± 6 ka (T3). The abandonment age of T2 in Kabaklık River located in “Section 2”is 34 ± 4 ka and the abandonment age of T3 is 11 ± 2 ka. The samples of the top terrace (T1) in Esnek River were dated to 38 ± 6 ka and 36 ± 5 ka. These two OSL ages indicate that the Esnek River began its incision about 37 ka ago since we observed a strath level below the T1 surface. The abandonment ages of T3 and T4 in the Esnek River are 26 ± 7 ka and 6 ± 2 ka, respectively (Fig. S2). OSL ages increase towards the upper terraces conforming to the morphostratigraphic positions of the terraces. The OSL ages derived from river terraces indicated that the highest river incision rates are from the Esnek River in “Section 5” (S5) representing the easternmost part of the range (range from 1.81 ± 0.26 to 1.35 ± 0.36 mm/a), and the lowest incision rates are from the Kabaklık River located in S2 (range from 0.55 ± 0.13 to 0.53 ± 0.09 mm/a). The Tabakhane River in S1 shows moderately higher incision rates (range from 1.06 ± 0.16 to 0.64 ± 0.09 mm/a) compared to the Kabaklık River in S2 (Fig. 3A, Fig. S5, Table S3).

### Catchment-wide erosion rates

The catchment-wide erosion rates vary from ~ 84 to ~ 530 mm/ka. Even though the number of erosion rate samples is not equal among the sections, the pattern of erosion rates broadly mimics the mean channel steepness index, river incision rates, and relief across the range (Fig. 5). The average catchment-wide erosion rates for each section show that the erosion rate is relatively higher in the eastern part of the range (S5). Catchments in S1, S2 and S4 are characterized by relatively lower erosion rates (Fig. 1C, Table S4).

### Topographic metrics of the source catchments

**Figure 4:**
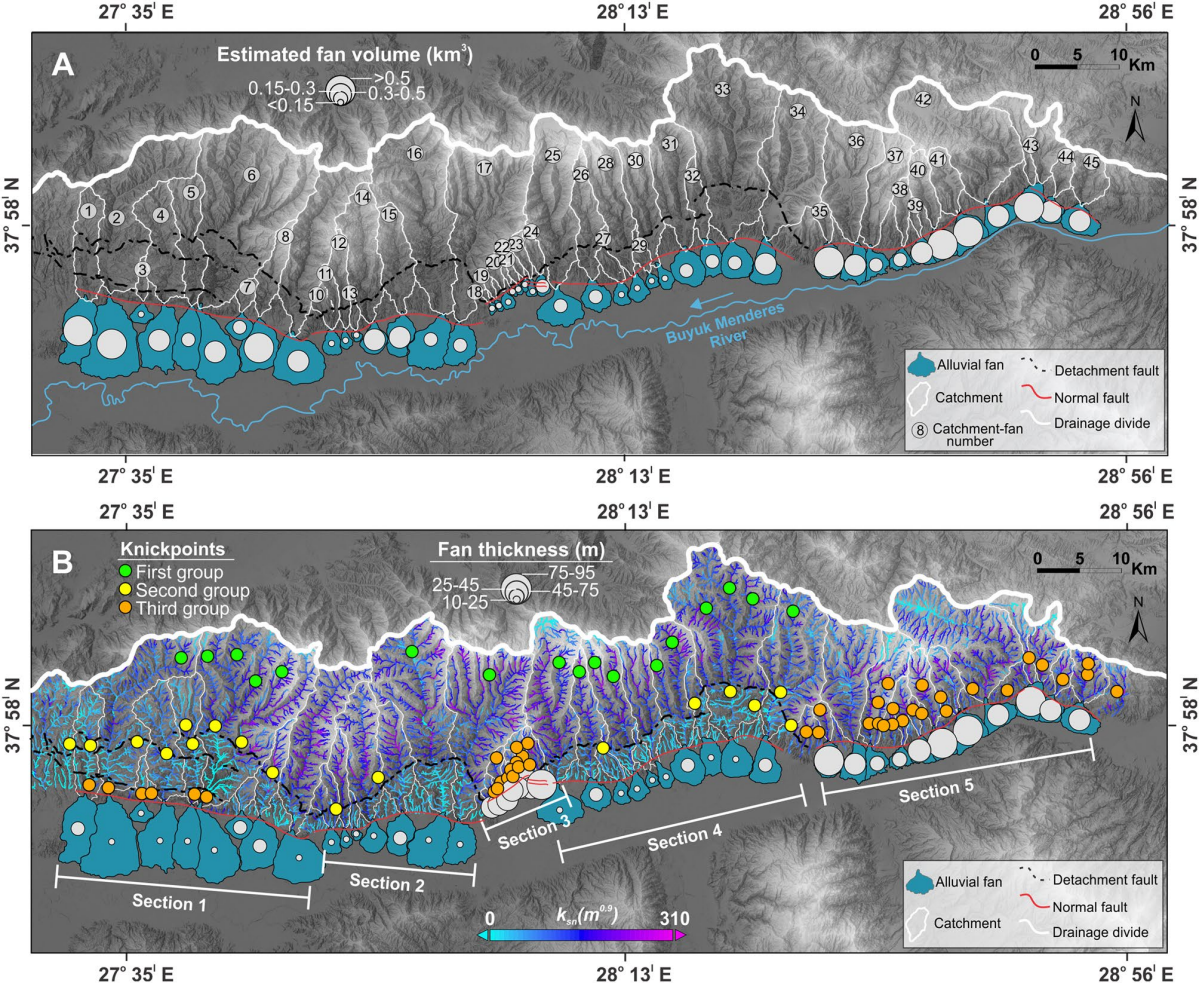
The volumes and thickness of alluvial fans along the southern mountain-front of the Aydın Range. (**A**) Estimated alluvial fan volumes with their catchments and (**B**) mean fan thickness with *k*_*sn*_ and knickpoints on footwall block.

The all mapped knickpoints show three different patterns the first group is found very close to the main drainage divide of the Aydın Range, and the second group of knickpoints largely match the lithological contacts and location of the Büyük Menderes Detachment Fault (Fig. 4B). The third group knickpoints which are slope-break knickpoints, are observed in S1, S3, and S5. High *k*_*sn*_ values (Fig. 3B) and spatial distribution of the third group knickpoints are clustered in S3 and S5 where there is relatively higher relief. Catchments for S2 and S4 have the lowest mean *k*_*sn*_ values of the range and do not have any third group knickpoints. The S1 with relatively lower elevation has third group knickpoints. The catchments for S3 and S5 are dominated by metamorphic and igneous rocks but the percentage of sedimentary rocks is slightly higher for catchments of S2 and S4 (Figs. 3C, 4B).

### Alluvial fan metrics

Alluvıal fan planimetric areas change from 1 to 36 km^2^. The estimated alluvial fan volumes vary between 0.02 and 1.01 km^3^. In general, the larger plan-view alluvial fans are represented by the greater volumes except in the eastern part of the range. Although alluvial fans in S5 have a smaller planimetric area compared to those in S1, their estimated volumes are higher (Figs. 3D, 4A). The mean alluvial fan thickness is 38 m across the southern mountain-front of the Aydın Range. The mean alluvial fan thickness is 57 m in S3 and 60 m in S5. The S1, S2, and S4 show lower alluvial fan thickness with a mean of 23 m relatively across the range (Figs. 3E, 4B). The average alluvial fan slope is 3.6°, and the mean alluvial fan slope of S3 with a higher gradient across the range-front is 5.4° (Table S5).

### Correlation between alluvial fans and their catchment parameters

For robustness, we used the Pearson's correlation coefficient to evaluate the strength of association between alluvial fans and their catchment parameters. A significant positive correlation was observed between catchment area—alluvial fan area (*r* = 0.69). In contrast, there is a weaker positive correlation between catchment area and alluvial fan volume (*r* = 0.19), relatively. We did not observe any meaningful correlation between alluvial fan volume and *k*_*sn*_ or catchment relief. Also, the correlation between alluvial fan volume and precipitation is very weak (*r* = 0.13). The alluvial fan thickness was significantly correlated with catchment *k*_*sn*_ (*r* = 0.52) and catchment relief (*r* = 0.48). The alluvial fan thickness shows a weaker relationship with catchment area (*r* = − 0.27) and precipitation (*r* = − 0.14), relatively (Fig. 2).

**Figure 5:**
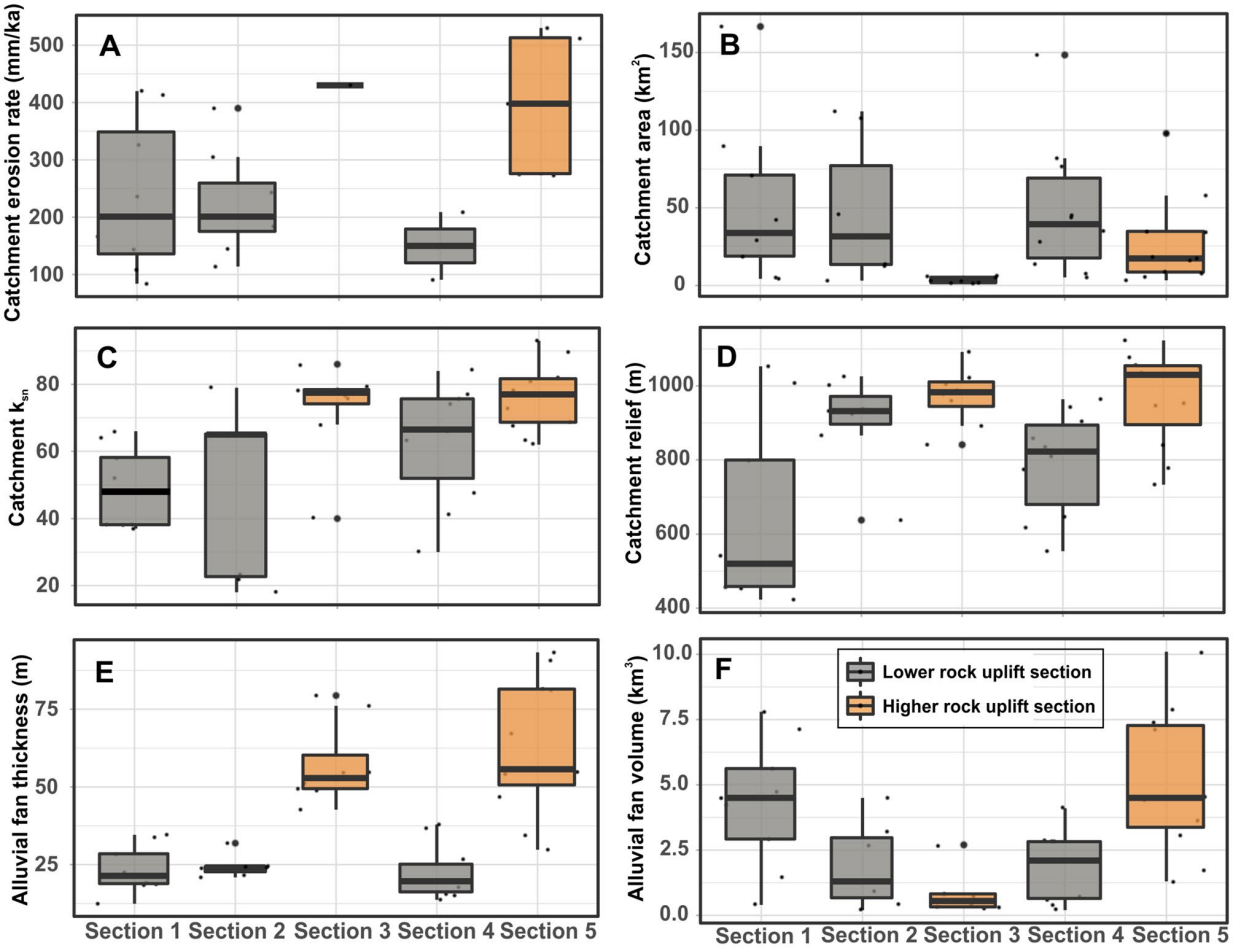
Boxplots showing the parameters of the alluvial fans and their catchments for each section. (**A**) Catchment-wide erosion rate, (**B**) catchment area, **(C)** the channel steepness index (k_sn_), (**D**) catchment relief, (**E**) the fan thickness, and (**F**) fan volume.

## Discussion

### Associations between 3D metrics of the alluvial fan and source catchment parameters

Pearson`s correlation coefficient indicated that catchment rainfall and the catchment area are not likely the dominant factors for alluvial fan volume or their thickness although the catchment area has a significant impact on the alluvial fan area (Fig. 2). The lowest precipitation values in S5 across the range are evidence that precipitation is not a first-order control on 3D morphometric features of alluvial fans in the study area (Fig. 3B, Fig. S10). Contrary to this, a strong correlation between the catchment area and the alluvial fan area implies that the catchment area is a major factor on the planimetric area of the alluvial fans in the study area. The variability of catchment-wide erosion rates in catchments with similar lithology also suggests that lithology is not a major control on variations in sediment flux to alluvial fans along the Aydın Range. The catchment lithology in S5, e.g., consists of more resistant igneous and metamorphic rocks compared to the relatively softer and more highly fractured sedimentary rocks of S2 and S4, yet S5 has greater alluvial fan volumes, relatively. Consequently, the weaker influence of precipitation, catchment size, and lithology on 3D morphology of the alluvial fans support the view that tectonics was mainly responsible for the three-dimensional morphology of the alluvial fans in the study area.

### Influence of the lower rock uplift on 3D morphology of alluvial fans

Our incision rates and river longitudinal profile analyses suggest a distinct differential rock uplif pattern along the southern flank of the Aydın Range. The low values of the incision rate, *k*_*sn*_, and relief suggest a lower rock uplift rate in S1, S2 and S4 relatively (Fig. 3F). The western part of the range (S1) is characterized by slightly higher incision rates compared to S2 and S4. The third group of knickpoints in lower elevation along the basin margin of the S1 suggests recent movement along the active high-angle normal faults. Fresh triangular facets and steep fault surfaces along the margin of S1 confirm this argument^44^. The combination of higher rock uplift and more friable Neogene sedimentary lithology in S1 may have given rise to higher sediment flux to alluvial fans than for catchments of S2 and S4 and enabled the creation of the most voluminous alluvial fans in the range margin. However, an insufficient rock uplift rate allows the sediment transport system on alluvial fans to overcome the effects of rock uplift. Due to the expansion of the alluvial fan areas towards the graben, the Büyük Menderes River is shifted its course towards the south.

**Figure 6:**
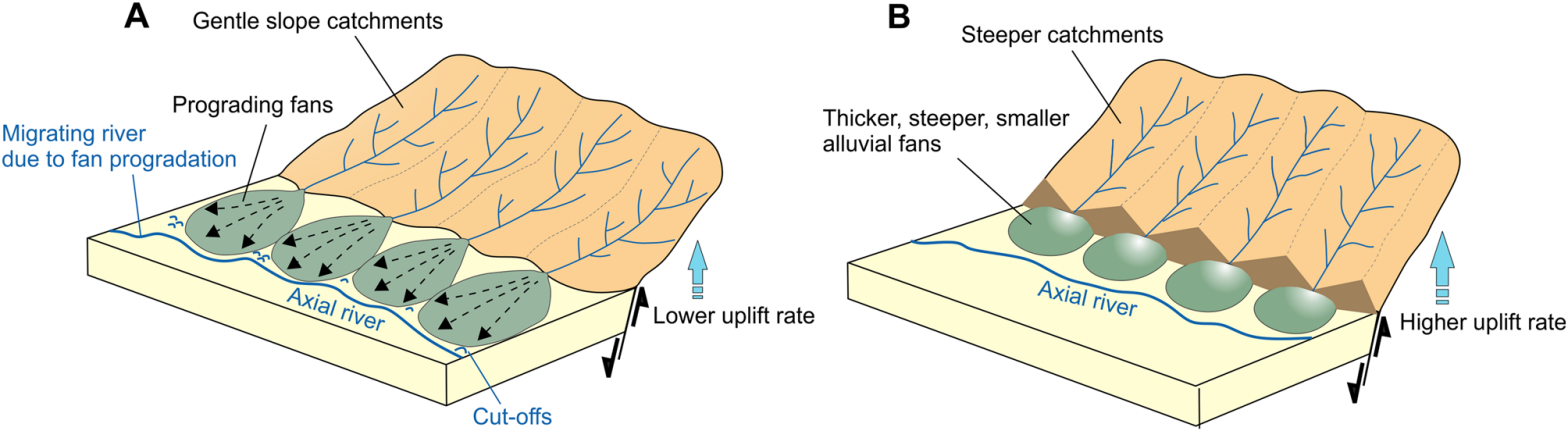
A conceptual model for the three-dimensional alluvial fan development with different uplift rates across a range front (modified from Attal^84^). (**A**) Relatively lower uplift rate gives rise to prograding fans due to insufficient sediment flux and accommodation space. This is an example of “S1”, “S2”, and S4 in the study area. (**B**) Higher uplift creates thicker fans with a small plan-view and steeper slope providing sufficient accommodation space and sediment flux from range. This is an example for “S3” and “S5” in the study area.

The S2 shows the lowest incision rates along the range. We did not observe any slope-break knickpoint in the south of the detachment fault for S2 and S4 which indicates that these sections have not been active as recently as S3 and S5 since the detachment fault activity. Indeed, previous studies found that the S4 has the lower fault slip rate across the range^42,43^. We think that the lower rock uplift rate likely decreased the erosion capacity of channels and accommodation space for the alluvial fans, hence allowing the formation of relatively lower fan thickness and gradient in these sections. As a result, the sediment transport on alluvial fans exceeds the rate of creation of accommodation space by rock uplift. Channels on alluvial fans disperse their sediment load further down, and the fan planimetric area expands into the Büyük Menderes Graben (Fig. 6A).

### Influence of the higher rock uplift on 3D morphology of alluvial fans

Clustering of high values of river incision rates, relief, and *k*_*sn*_ confirms that S3 and easternmost parts (S5) are the zones of higher uplift (Fig. 3E, Fig. S11). In particular, variations in elevations of the third group knickpoints represented by tectonic activity following Büyük Menderes Detachment Fault activity reflect nonuniform background rates of rock uplift in S3 and S5. Our results agree with Barbot and Weiss^43^ that normal faults in the eastern part (S5) of the Aydın Range have the highest slip rates in the range. Also, Altunel et al.^42^ indicated that normal faults in S3 have higher slip rates than in S2 and S4. We argue that the high rock uplift rate in the source footwall accelerated erosion and transport of sediments fluvially, increasing the slopes of the channels feeding the alluvial fans. The relatively higher pattern of catchment-wide erosion rates in S3 and S5 likely confirms this view. The reason why previous^24,28^ studies did not find any relationship between tectonics and catchment-wide erosion is probably that these earlier studies focused mostly on the western part of the range. Our new catchment-wide erosion data from the missing parts of the range have indicated that catchment-wide erosion mostly shows a pattern compatible with tectonic uplift across the range. High rock uplift rates in S3 and S5 are probably due to the rapid development of accommodation space and the sufficient volume of sediments to fill this space. Since the rock uplift rate exceeds the rate of erosion, alluvial fan thickness increases with a steeper gradient (Fig. 6B).

### Importance of three-dimensional morphology of the alluvial fans

Since the 1960s, when attention began to focus on the morphometric characteristics of alluvial fans, it has been hypothesized that the fan area represents its volume^19,20,57^, but this assumption has not been tested in a tectonically active natural place. Analysis of 45 alluvial fans in a tectonically active region can support a general theory^55,56^ that the power law ratio of fan area to fan volume is about 1:1^22^. Based on the fact that the fan area adequately defines the fan volume, a high correlation is also assumed between the fan volume and the catchment area. In contrast to the previous studies^5,22^, we found a relatively weaker correlation between fan volume and catchment area. The fact that the catchments to the east of the Aydın Range have almost similar fan volumes despite being relatively smaller than the catchments to the west can be explained by the high erosion caused by the higher uplift (Fig. 5). Therefore, we suggest that relationships between fan volume-catchment areas must be considered in the context of the local tectonic setting. Additionally, using as a marker the planimetric area of alluvial fans for tectonic implications may cause inaccurate interpretations. For example, alluvial fans in relatively higher rock uplift zone (“Section 3”) have the smallest planimetric area across the range. On the other hand, the alluvial fans in “Section 2” and “Section 4” have smaller planimetric areas compared to “Section 5”, but they are represented by the lower rock uplift rate, relatively. Accordingly, the planimetric area of alluvial fans does not show any meaningful pattern with the rock uplift rate. The thickness of the alluvial fans in the study area mimics the variations in the pattern of the uplift rate. If we consider the relief and *k*_*sn*_ as a proxy of tectonics in the footwall block, fan thickness responds to tectonic uplift according to the Pearson correlation coefficient. Therefore, our data imply that the alluvial fan thickness rather than the alluvial fan volume and planimetric area of the alluvial fans respond more sensitively to the tectonic uplift of the footwall block which is consistent with earlier modeling studies^13,14,54^.

Unfortunately, there is no direct study on the alluvial fans in the study area to compare with our results. We compare our results with earlier modeling and field-based studies on a global scale. Our results conceptually conform to the fan development proposed by previous modeling studies^13,14^ suggesting that alluvial fans are thicker in regions of more significant tectonic uplift because the higher uplift provides more vertical accommodation space in the hanging wall for the sedimentary flux to fans compared to lower uplift rate^54^.

## Conclusions

The main point of the analysis in this study is that in an extensional tectonic setting where subsidence is not uniform, relative three-dimensional morphology is largely controlled by the spatial distribution of rock uplift or subsidence rates and bears little direct relationship to the physical characteristics of the source area. This study highlights the role of differential rock uplift on the three-dimensional morphology of alluvial fans which has significant implications for understanding how 3D morphometric features of alluvial fans record the tectonic perturbation along a range in an extensional region. In contrast to some earlier field-based fan morphometric studies, we suggest that 3-D morphological knowledge of alluvial fans is needed to make tectonic inferences. Specifically, our data indicated that fan thickness rather than fan area or fan volume reacts more sensitively to the uplift in the source footwall block. Consequently, these findings indicate that a combination of volumetric and planimetric measurements of alluvial fan systems can be used to elucidate the pattern of relative tectonic activity where the impact of precipitation and lithology are controlled and can constrain global alluvial fan development models that operate at this scale.

## Methods

### Red relief image map

TanDEM-X (12.5 resolution) was used as a base map for mapping and all topographic analyses in this study. Firstly, we created a Red Relief Image Map (RRIM) to map and identify the modern alluvial fans and river terraces. Özpolat et al.^44^ showed that the RRIM is a highly useful technique to map the landforms such as alluvial fans, river terraces, and landslides. The RRIM visualization technique, as proposed by Chiba et al.^58^, employs the multiplying three layers namely negative openness, positive openness, and the topographic slope. The RRIM is calculated in the following formula:

1 $$I=({O}_{p}-{O}_{n})/2$$

The negative openness (On) and positive openness (Op) are angular measures of the topographic convexity and concavity, respectively^59^. Negative openness shows higher values such as valleys, craters, and landslides while positive openness represents higher values such as crests and ridges. The *I* value emphasizes concave and convex landforms with a grey gradation. The red color is preferred in the topographic slope since it is the richest tone for human eyes (Fig. S1).

### OSL dating of the river terraces and incision rates

From west to east across the Aydın Range, we mapped at least four terrace surfaces above the modern Tabakhane River, three terrace surfaces above the modern Kabaklık River, and four terrace surfaces above the modern Esnek River (Fig. 1C). Eight samples were collected from three different river terrace staircase locations. To determine the maximum ages of terrace abandonment, we focused on sampling from the uppermost terrace surfaces. Thus, we obtained the longest terrace incision history. The optically stimulated luminescence (OSL) dating method was applied to obtain the abandoned ages of the river terrace staircase. Samples from terrace sites were selected based on visual observations of homogeneity of the sediment to ensure uniform gamma irradiation. Because alluvial and fluvial processes consist of energetic processes, they provide poorly bleached OSL signals. Hence, samples were collected from silt lenses that were deposited in less-energetic processes for OSL dating. We kept a minimum distance of 1 m below the terrace surface. OSL samples were collected by using hammering opaque steel tubes (45-cm-diameter by 35-cm in length) into vertical exposures. To determine the dose rate, samples were collected from sediment surrounding the equivalent dose sampling.

The OSL samples were analyzed at the Luminescence Dating Laboratory, Ankara University, Turkey. All sample mineral separation and chemical protocols in the laboratory were carried out under subsided red-light conditions. All sample mineral separation and chemical protocols in the laboratory were carried out under subsided red-light conditions. Samples were undergone by obeying conventional luminescence dating laboratory procedures and protocols^60^. For this purpose, firstly after carefully extracting from opac sampling tubes, the sands were sieved to obtain the 90–140 μm particle size fraction. Subsequently, it was treated with 10% hydrochloric acid (HCI) to remove carbonates and 35% hydrogen peroxide (H_2_O_2_) to remove any organic components overnight. After drying, a heavy-density liquid is used to separate quartz minerals. Lastly, the quartz was etched with 40% hydrofluoric acid (HF) for 45–60 min to remove the outer surfaces of the grains in which alpha dose was dominant and then with 10% hydrochloric acid (HCI) for 1–2 h to dissolve precipitated fluorides. For measurements of OSL, the quartz grains were prepared mass of ~ 2 mg aliquots were prepared by mounting the material on stainless-steel cups/disks. The aliquot size was 5 mm which corresponds to roughly 500–1000 mineral grains mounted on stainless steel discs as monolayers within the selected size range between 90 and 140 μm. The quartz mineral quality was controlled by the “IR check” method after all chemical steps by stimulating quartz grains with IRSL at 50 °C. In case any felspathic signal contribution is observed, one more IR stimulation step at 50 °C 100 s is added between preheating and OSL stimulation steps to eliminate felspathic contribution in quartz luminesce emission^61^.

The equivalent dose (ED) measurements were performed using Risø TL/OSLDA- 20 reader equipped with a ^90^Sr/^90^Y beta source (delivering dose rate 0.110 ± 0.004 Gy/s) for artificial doses and a 9635QA photomultiplier tube for luminescence emission detection. For the detection of OSL, a 7.5 mm Hoya U-340 filter (~ 340 nm, FWHM ~ 80 nm) was used. The equivalent dose calculations were carried out based on fast component OSL dating. Equivalent dose (ED) measurements were determined using the conventional single-aliquot regenerative-dose (SAR) protocol^62^ (Fig. S6, Table S1). At least 24 aliquots have been used for each sample, obeying statistical approximation rules in the radial distribution of EDs. The Central Age Model (CAM) was used towards assessing the corresponding ages. Figures S7 and S8 displays a typical dose–response graph for the corrected luminescence signal, Li/Ti of a selected aliquot. Concentrations of the radioactive isotopes U, Th, K, and Rb of the samples were measured at Acme Analytical Laboratories to use annual dose rates. The water saturation content in the samples was calculated using an automatic moisture analyzer, yielding a value of 30 ± 5% in all samples (Table S1).

Cosmic radiation contributions were calculated using geographical conditions of the sample such as the sampling depth, elevation, and location as indicated by Olley et al.^62^ and Prescott and Hutton^60,63^. The luminescence ages were calculated both using the online Dose Rate and Age Calculator for trapped charge dating (DRAC) and Luminescence Dose and Age Calculator (LDAC)^61,64–66^. Summarizing all the related data for the luminescence dating evolution is given in Table S2.

The long-term river incision rate was calculated through a combination of the incision depth data with the available OSL ages obtained from the terrace surfaces (Table S3). The longest river incision rates were used to evaluate the pattern of relative rock uplift in this study.

### Catchment-wide erosion rates based on ^10^Be

We collected riverbed sediment samples from eight streams that flow into BMG along the southern flank of the Aydın Range to determine ^10^Be concentrations. Most samples in the range are from rivers that exclusively drain igneous and metamorphic rocks. Each sample was collected from several points along the respective stream over 15–30 m. Samples were initially wet-sieved to 1–0.25 mm grain size in the field and later at the İTÜ/Kozmo-Lab at Istanbul Technical University (https://www.kozmo-lab.itu.edu.tr/en).

The remaining sample processing and AMS target preparation were completed at CRISDal Lab, Dalhousie University, Canada. Mineral separation of quartz using combinations of heavy liquids, froth floatation, Frantz magnetic separation, and partial digestions in aqua regia or HF ultrasonic baths continued until abundances of native Al in the quartz concentrate were below 100 ppm Al and Ti (as determined on 0.5 g of sand aliquots using ICP-OES) and the quartz concentrate appeared pure under an optical microscope. Type 2 water (RO with some deionization) was used for the previous steps and Type 1 (18.2 MOhm) deionized water was used for all subsequent steps including acid cleaning of any labware and target holders, using an Elga Centra-R60 water purification system with continuous recirculation to the dispensing sites, where there are additional mixed-bed resin columns to ensure 18.2 MOhm, plus an extra milli-Q boron filter and final milli-Q mixed-bed resin column for the ^10^Be lab. Following the quartz purification procedure, we removed an additional 30–35 wt% of the quartz to remove any meteoric ^10^Be still attached to the surface or microfractures in the quartz grains^69^. We spiked the quartz (most masses were 40 g, but as low as 20 g in samples with insufficient quartz) with ~ 210 µg Be, using the Be carrier “Be Carrier B11 2020-03-06” which was produced at CRISDal from phenacite sourced from the Ural Mountains, with an ICP-OES-measured average Be concentration of 685 ± 21 µg/ml and density of 1.013 g/ml. We digested the mixture with the minimum needed volumes of concentrated trace-metal grade aqua regia, HF, and perchloric acid. The cake was dissolved and brought up to 100 ml in 2% ultrapure nitric acid, and we extracted a 5 ml aliquot to ensure that the concentration of native Be in the quartz was negligible compared to the spike. To extract the Be from the solution, we used 5 ml of AG-50W-X8-200-400 mesh and a CRISDal elution procedure that was calibrated with elution experiments for a similar quartz mass and major element composition. The separated Be chloride solutions were centrifuged, converted to Be (OH)_2_ using ammonia gas, and then calcined to BeO with a bunsen burner (3 min minimum). The targets were massed, carefully powdered in their boron-free quartz vials in a static-free glovebox, then mixed well with Nb metal, packed tightly to the optimal height for Cs-sputtering in thoroughly cleaned stainless steel target holders, and then kept in a desiccator until couriered. All AMS measurements were completed at the Centre for AMS at Lawrence Livermore National Lab (CAMS-LLNL). The AMS standards used for normalization of the sample results were 07KNSTD3110 with ^10^Be/^9^Be of 2.85 × 10^–12^ at/at. In this experiment, the carrier produced a process blank ^10^Be/^9^Be of 8.0 ± 1.8 × 10^–16^, which was in the range of other process blanks using this carrier but with different masses of quartz (4–10 × 10^–16^). Sample precisions averaged 3.6% and ^9^Be currents averaged 10 µA. The total analytical uncertainty reported includes the AMS precision, uncertainty in the carrier concentration (3%), and the small (< 1%) error contributed by the blank subtraction.

Catchment-wide erosion rates from ^10^Be concentrations were calculated with the CRONUS-Earth online calculator (http://hess.ess.washington.edu)^68^ using the primary calibration data set of Borchers et al.^69^ and the time-invariant production rate scaling model^70,71^. The new erosion rates are integrated with 16 previously published catchment-wide erosion rates^24,28,83^ to examine spatial variability. We used the same cosmogenic production rate and scaling schema as the earlier studies in the study area to make an easy comparison. For calculating the erosion rates, we used a bedrock density of 2.5 g/cm^3^ and 2.2 g/cm^3^ for catchments in Neogene sedimentary rocks. We used the mean catchment elevation. We did not take into account a topographic shielding factor for erosion rate calculation since DiBiase^72^ recently showed that this correction is not required (Table S4).

### Channel steepness index and knickpoint extraction

The channel steepness index (*k*_*sn*_) and knickpoint distribution were analyzed to capture information about the spatial pattern in the rock uplift along the southern flank of the Aydın Range. River longitudinal profile analysis relies on power-law relationships between local channel slope (*S*) and the contributing upstream drainage area (*A*):

2 $$S={k}_{s}{A}^{-\theta }$$

where *k*_*s*_ refers to the channel steepness and *θ* designates the concavity index^73^. We produced a mean value from all concavity indices which is a concavity of 0.43 ± 0.3 (2σ) for streams that drain the southern flank of the Aydın Range based on the integral analysis of Perron and Royden^74^. For a suitable reference concavity *θ*_*ref*_, the *k*_*sn*_ is expressed as:

3 $${k}_{sn}=S {A}^{{\theta }_{ref(0.43)}}$$

The obtained values are averaged for each catchment. To produce a normalized channel steepness index (*k*_*sn*_) and concavity index for the study area, we used the ChiProfiler tool^75^ that utilizes TopoToolbox 2^76^ in MATLAB based on an integral approach called χ-analysis^77^. The normalized steepness indices were produced for channel segments of 1 km length for streams draining the Aydın Range.

Slope-break knickpoints are related to active or ancient tectonic forcing and are valuable topographic features to evaluate the tectonic deformation pattern^53,78^. We extracted the “slope-break knickpoints” with different *k*_*sn*_ values in upstream and downstream longitudinal profiles on log–log area-slope diagrams using ChiProfiler in MATLAB^75^ (Figs. S9, S10). All verified slope-break knickpoints that are in contact with different rock types were excluded from further interpretation in this study. Finally, the spatial distribution of these knickpoints serves as a guide to evaluating the pattern of rock uplift^53,77^. Geophysical relief was produced with a 500 m window size based on Small and Anderson^79^ and the mean catchment slope was calculated from TanDEM-X.

### Fan volume estimation

Modern computational techniques based-Digital Elevation Model (DEM) can measure the volume precisely considering the present-day topography of a surface^80–82^. Calculating volume using DEM has a simple algorithm which is an area of a pixel (p) multiplied by the value (h) of a respective pixel that contains the depth information of the surface in each pixel. *A* is the cell size, and *b*_0_ represents the base level elevation for each fan. The total volume (V_*f*_) is a summation of each pixel area multiplied by the respective pixel value as shown in the following formula:

4 $${V}_{f}=A\sum_{i=1}^{n}Pi*({h}_{i-}{b}_{0})$$

We focused on the visible conical alluvial fan volumes enclosed between the base surface and the present-day topography of the alluvial fan surface. To calculate the volume, we determined a base level which the volume is referenced from. This base level for a fan is a reference elevation from where the surface height was calculated. The base surface of the alluvial fans was approximated using the elevation immediately around the toe of each alluvial fan using multiple transects and contours. After a base level surface was determined, the elevation of this surface was used to get the height of a pixel from the reference base level. The pixel values were subtracted from the base level elevation. However, since subsurface topographic data is not available, we need the following assumptions: (1) the base of the fan is the almost horizontal surface (2) a vertical abutment against the mountain-front from extending down. These assumptions may result in an overestimation of fan volume. In reality, the alluvial fan volume calculations based-DEMs are gross estimates and are simplifications if precise subsurface topographic data is lacking. Since we apply assumptions for all alluvial fans one by one, we do not think that the volume estimates will affect the interpretation in terms of the spatial pattern. Finally, all fan volumes were normalized to their areas to obtain the mean thickness for each alluvial fan.

## Supplementary Information

Supplementary Information.

## Data Availability

All data generated or analyzed during this study are included in this published article [and its supplementary material files].
