# Supplementary material for: Three-dimensional control of alluvial fans by rock uplift in an extensional regime: Aydın Range, Aegean extensional province
Source: Sci Rep. 2022 Sep 12;12:15306. doi: 10.1038/s41598-022-19795-0 (PMC9468007; doi:10.1038/s41598-022-19795-0)
Supplement: Supplementary file 1 — Supplementary Information. [file 41598_2022_19795_MOESM1_ESM.pdf]

Supplementary Material for

Three-dimensional control of alluvial fans by rock uplift in an extensional regime: Aydın Range, Aegean extensional province

Emrah Özpolat[1,2*], Cengiz Yıldırım[2], Tolga Görüm[2], John C. Gosse[3], Eren Şahiner[4], M. Akif Sarıkaya[2], and Lewis A. Owen[5]

[1]Department of Geology and Environmental Science, University of Pittsburgh, Pittsburgh, PA 15260, USA

[2]Eurasia Institute of Earth Science, Istanbul Technical University, Istanbul, 34469, Turkey

[3]Department of Earth and Environmental Sciences, Dalhousie University, 1355 Oxford Street, Halifax, Nova Scotia B3H 4R2, Canada

[4]Institute of Nuclear Science, Ankara University, Ankara, 06800, Turkey

[5]Department of Marine, Earth, and Atmospheric Science, North Carolina State University, Raleigh, NC 27695, USA

[*]Corresponding author: emrahozpolat@pitt.edu

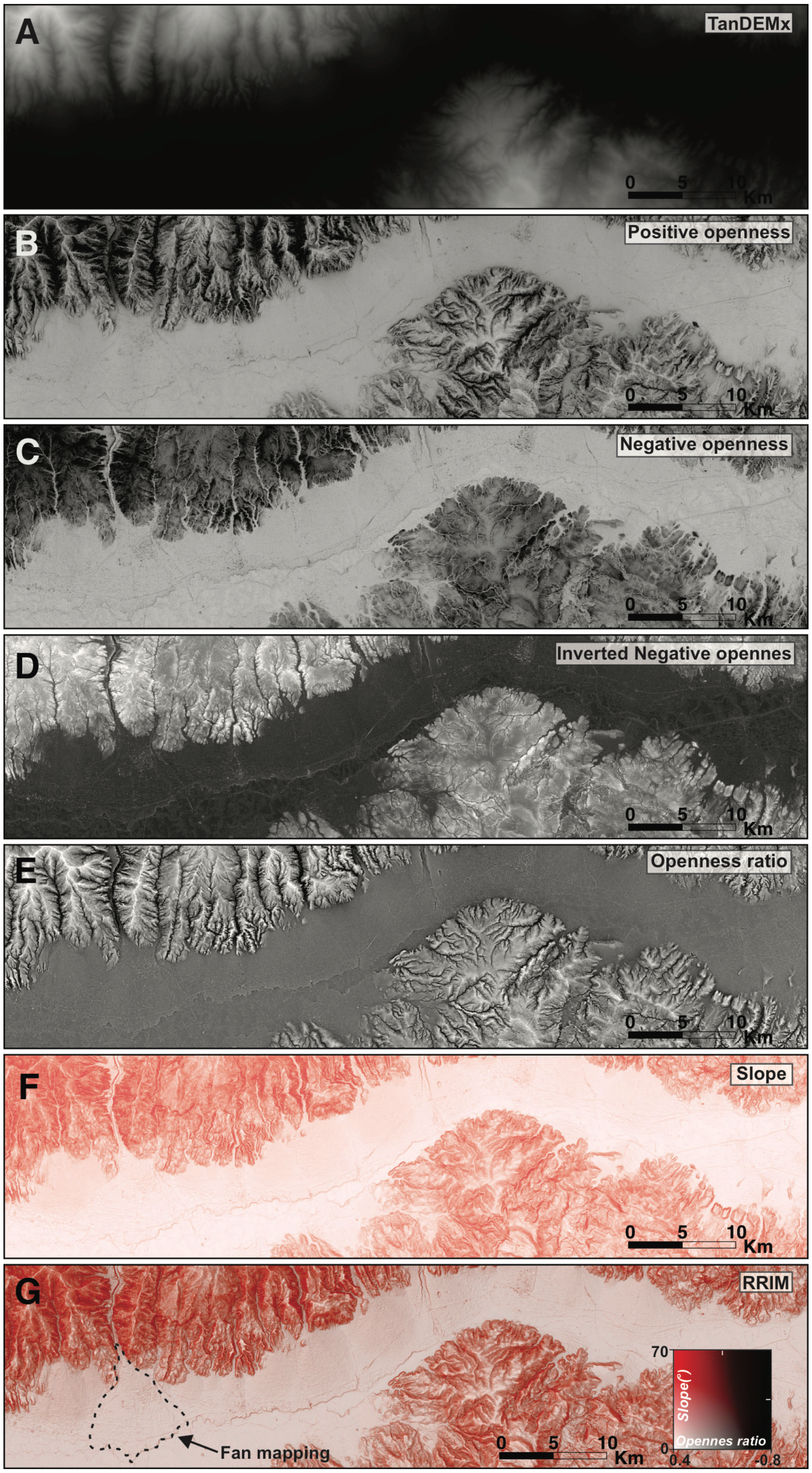

Figure S1. Red Relief Image Map (RRIM) production steps (from the eastern part of the southern mountain front of Aydin Range). A) TanDEM-X Digital Elevation Model, B) positive openness, C) negative openness, E) openness ratio between positive and negative openness, F) slope map derived from TanDEM-X, G) Red Relief Image Map and mapping of an alluvial fan in Section 5.

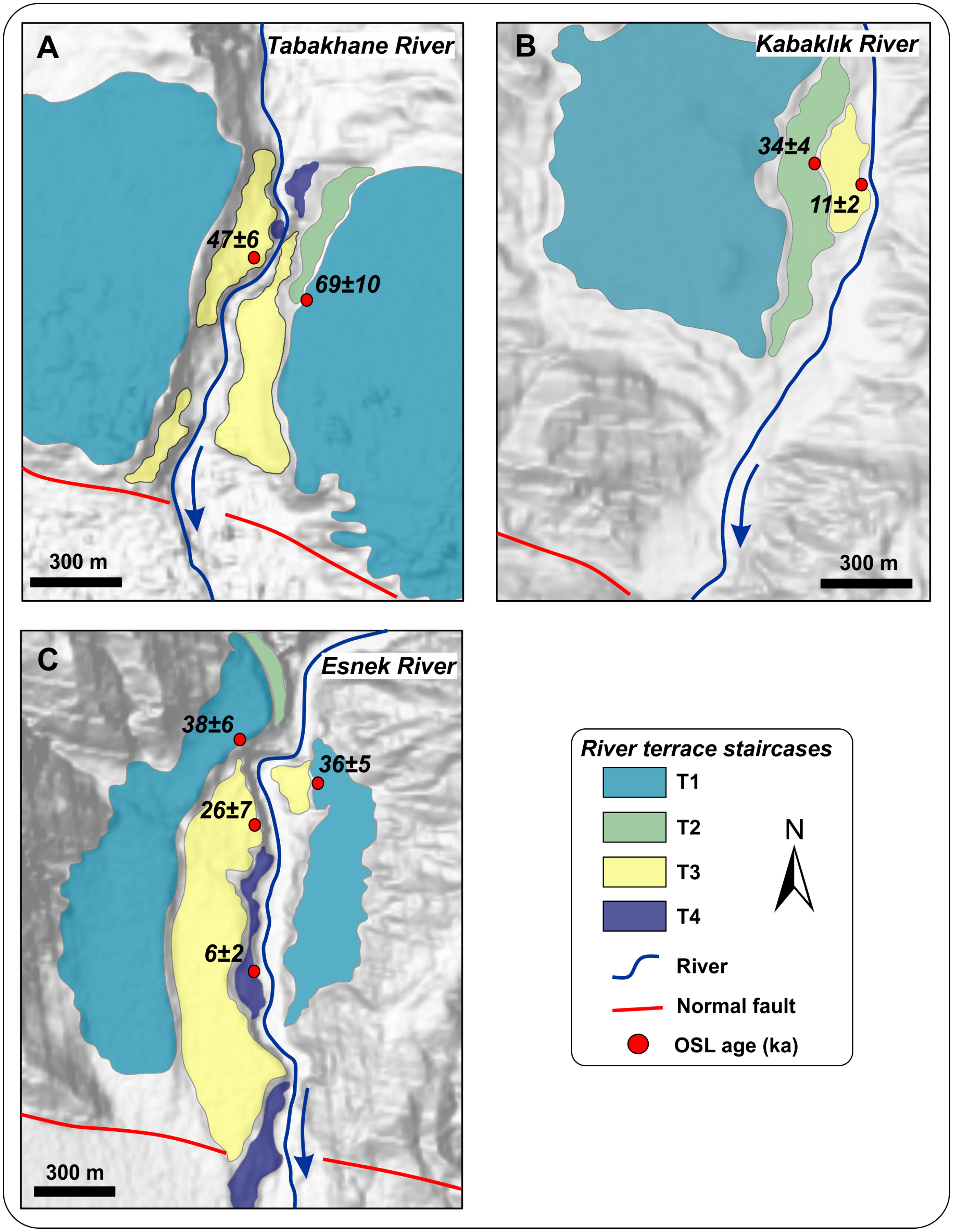

Figure S2. Terrace staircases of Tabakhane (A), Kabaklık (B), and Esnek River (C) on TanDEM-X digital elevation model.

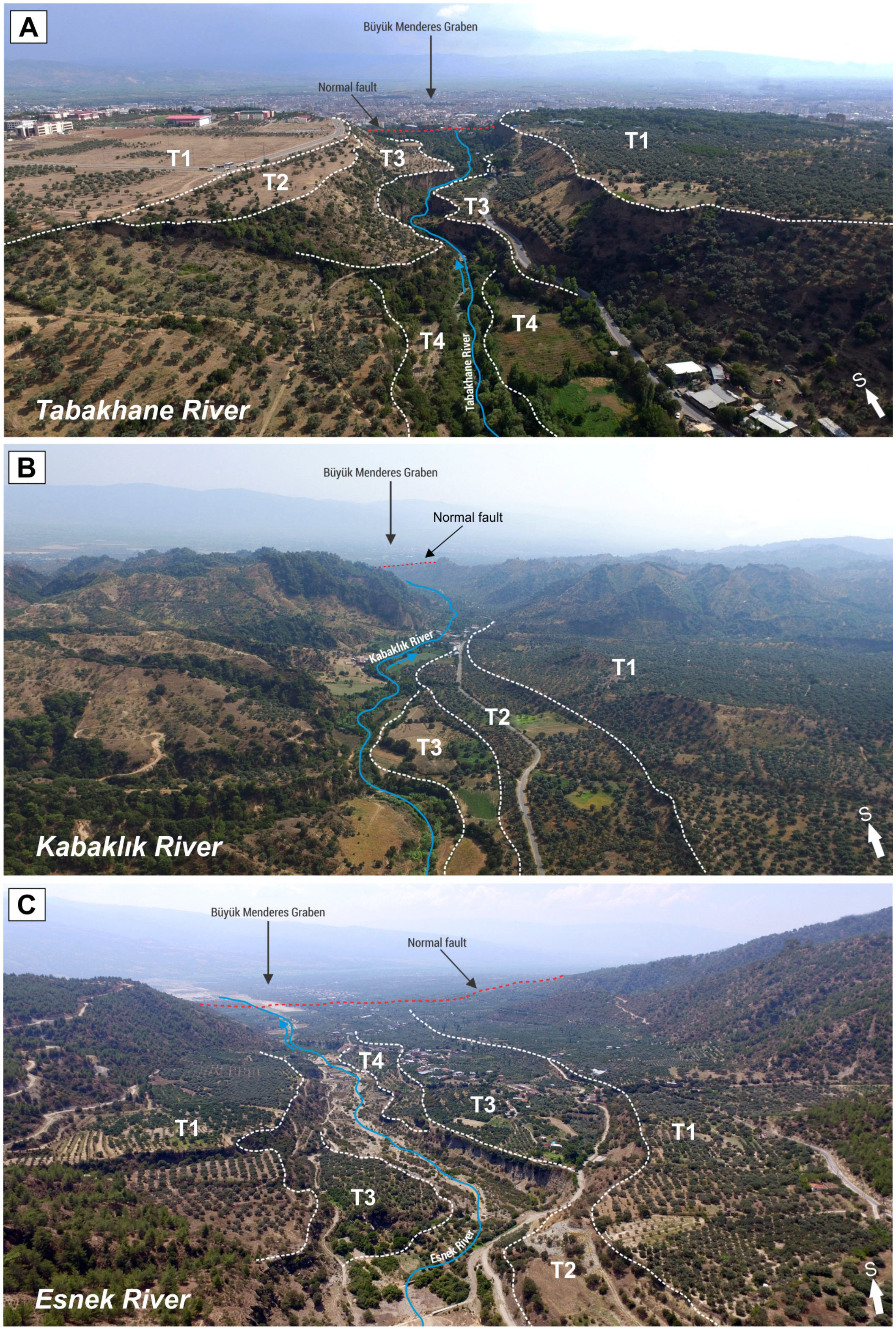

Figure S3. Oblique drone pictures of the river terrace staircases. (A) Tabakhane, (B) Kabaklık, and (C) Esnek River. See Figure 1C for terrace locations.

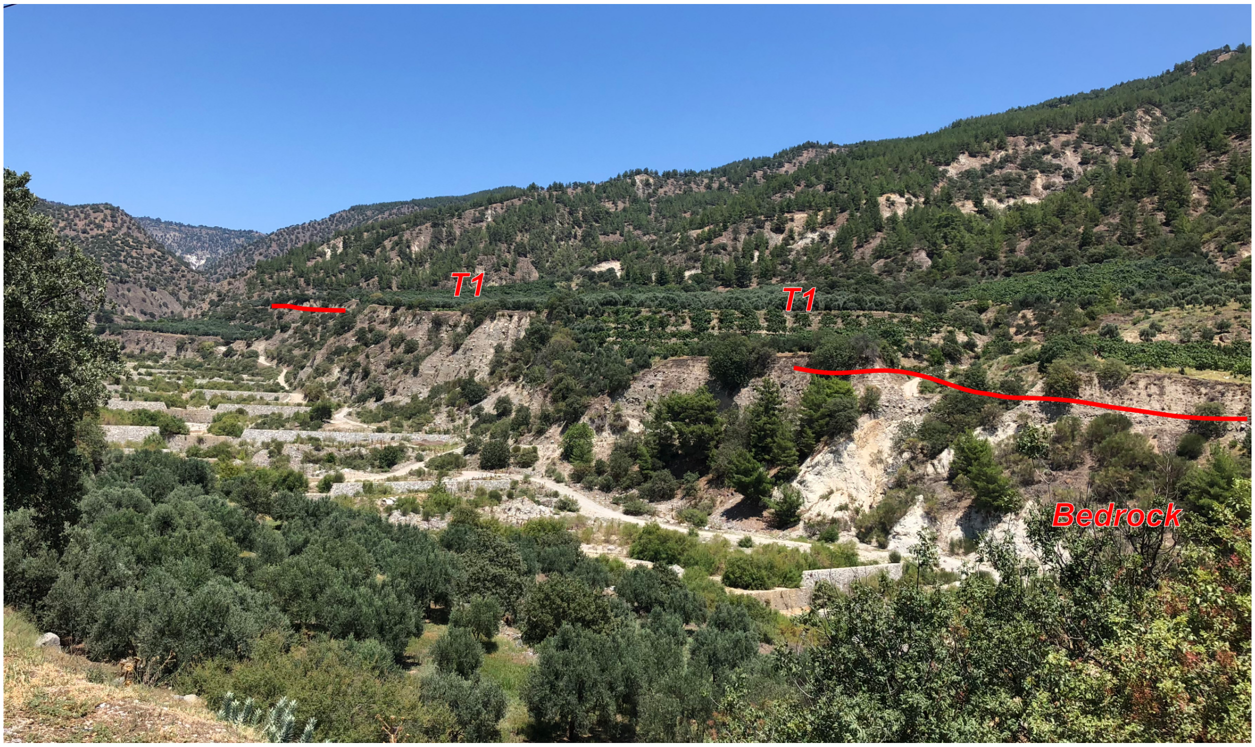

Figure S4. Oblique drone picture of the T1 surface, strath level, and bedrock in the Ensnek River terraces.

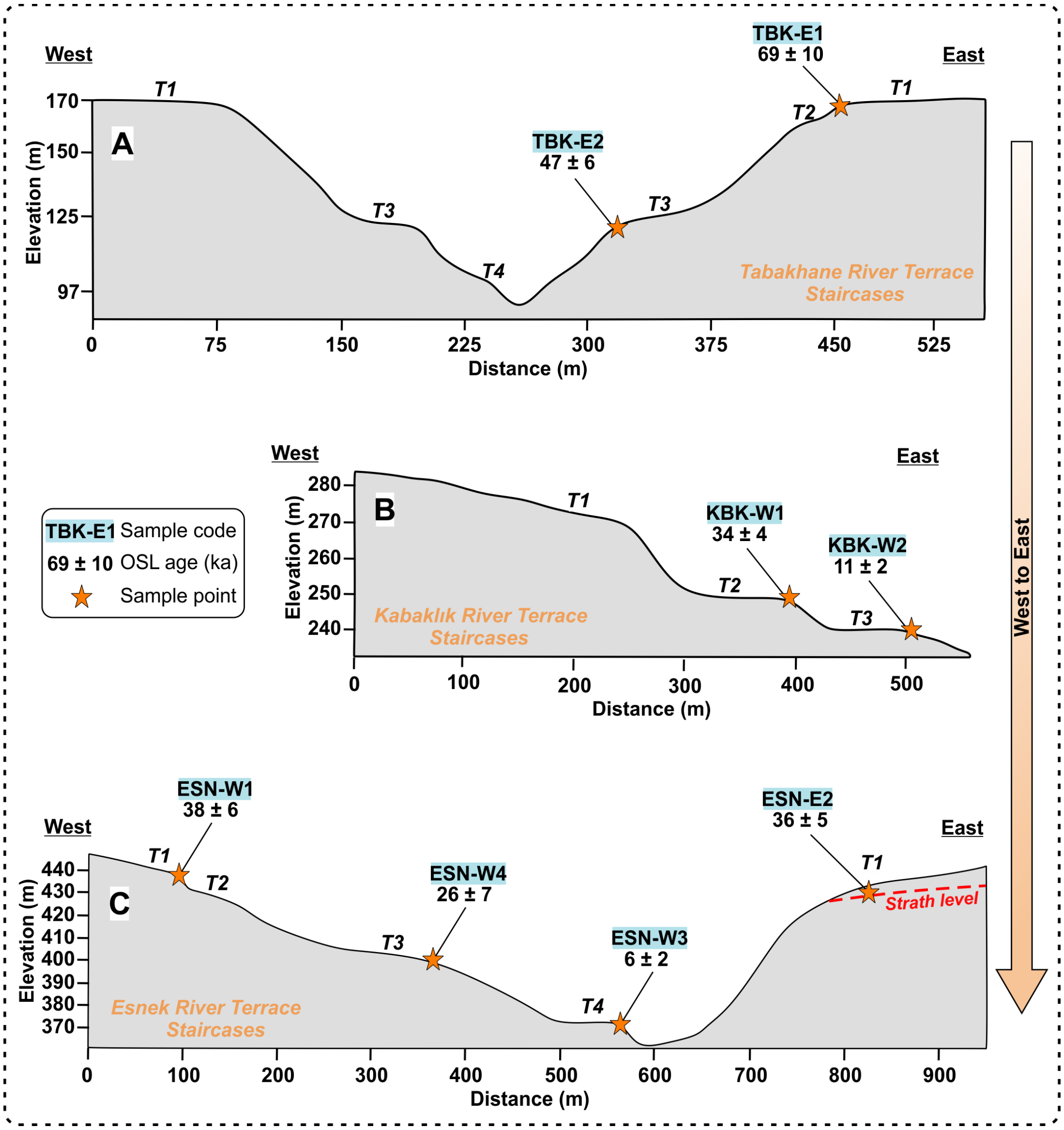

Figure S5. Transverse section of the river terrace staircases. (A) Tabakhane, (B) Kabaklık, and (C) Esnek River. See Figure 1C for terrace locations.

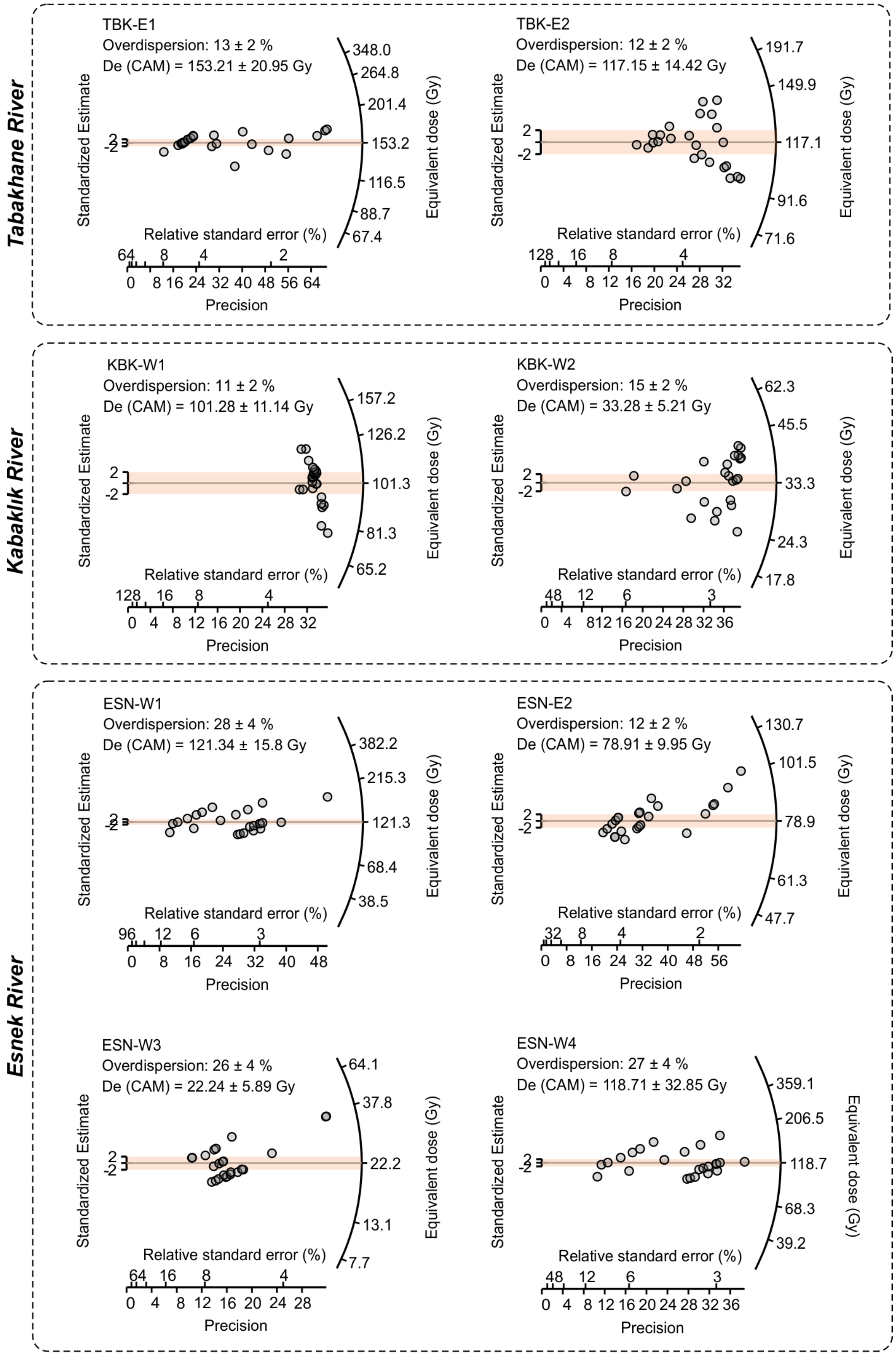

Figure S6. The radial plot for EDs yielded (<2σ) after measuring 24 aliquots for each sample. The values with associated individual aliquots (The same analyses were performed for all samples).

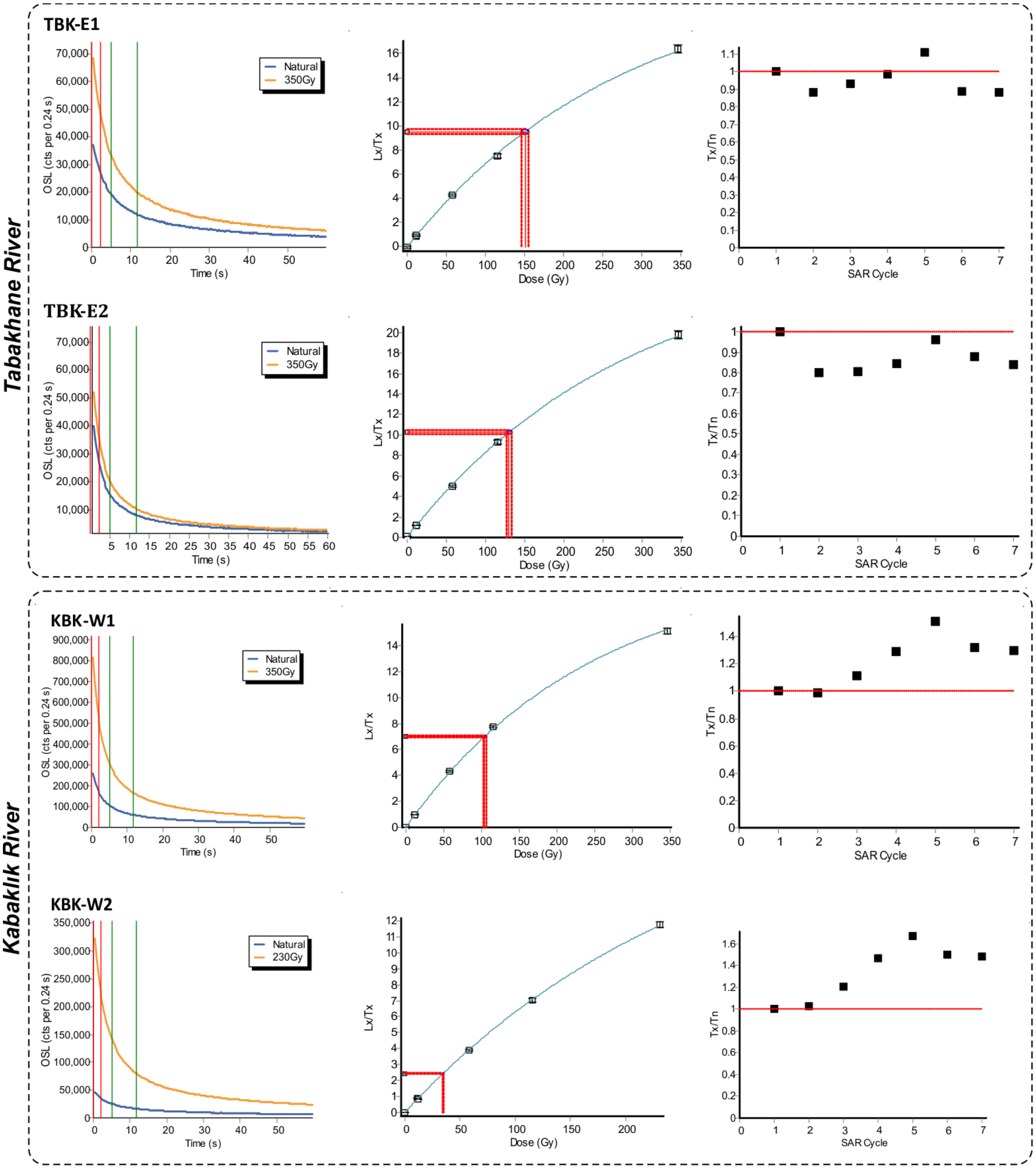

Figure S7. The OSL shine-down curves for the natural and regeneration doses, typical dose-response for the OSL signals (the vertical axes show the corrected OSL signal and the horizontal axes the laboratory radiation dose in Gy and the red dotted line also indicates the ED), and the variation of Lx /Tx ratios during SAR-OSL cycles.

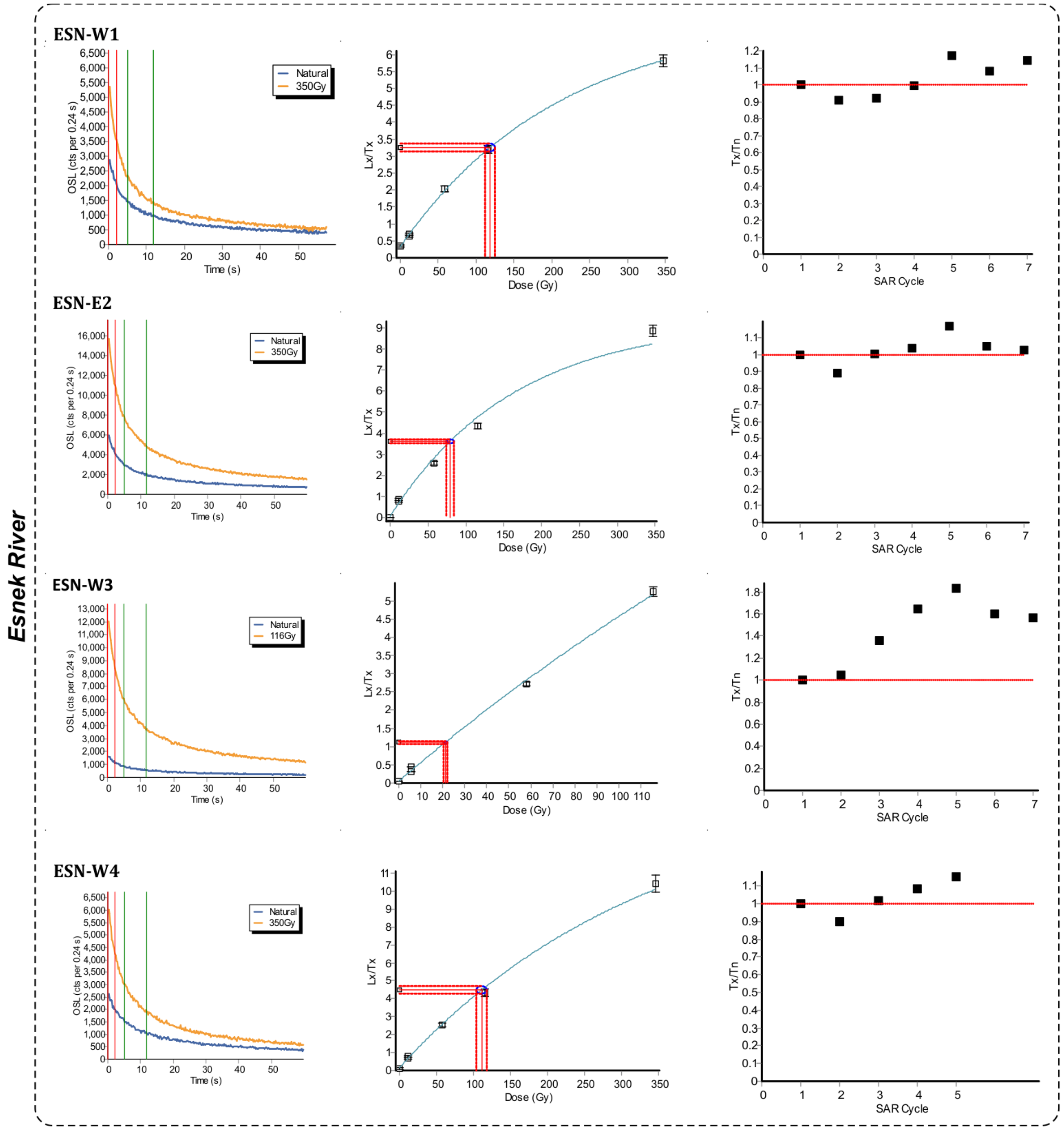

Figure S8. Continued.

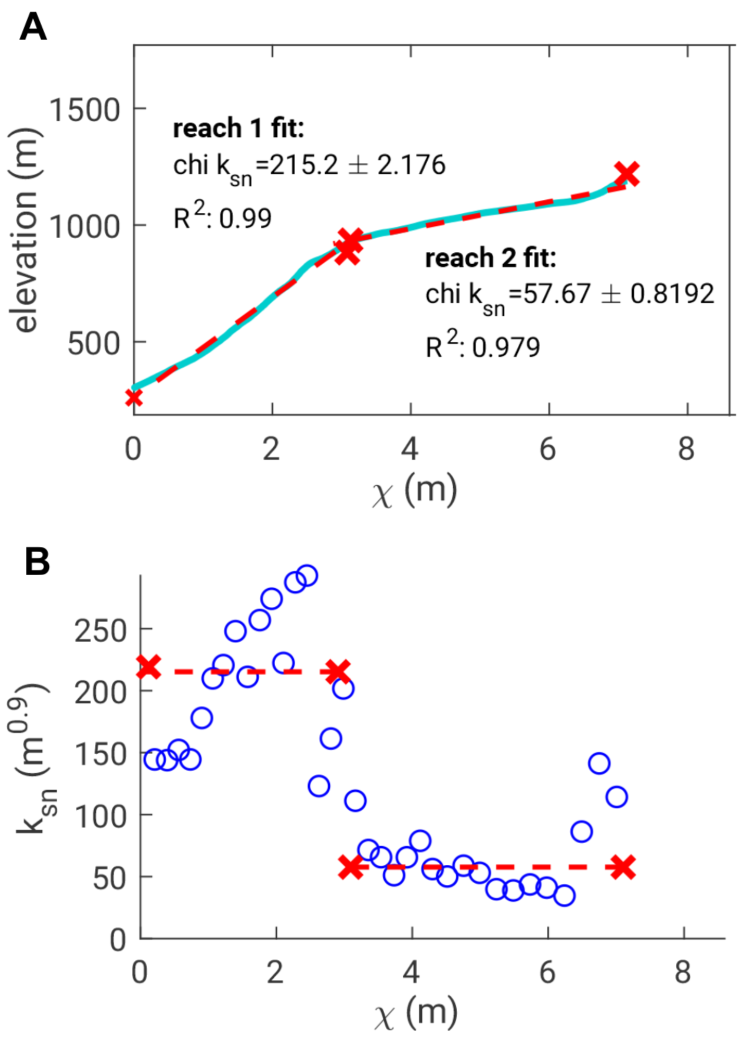

Figure S9. An example of slope-break knickpoint is associated with the tectonically induced knickpoint (trunk river of basin no.40). A) k_{sn} value of different reaches and B) k_{sn} versus χ (m) to detect the knickpoints on a river longitudinal profile.

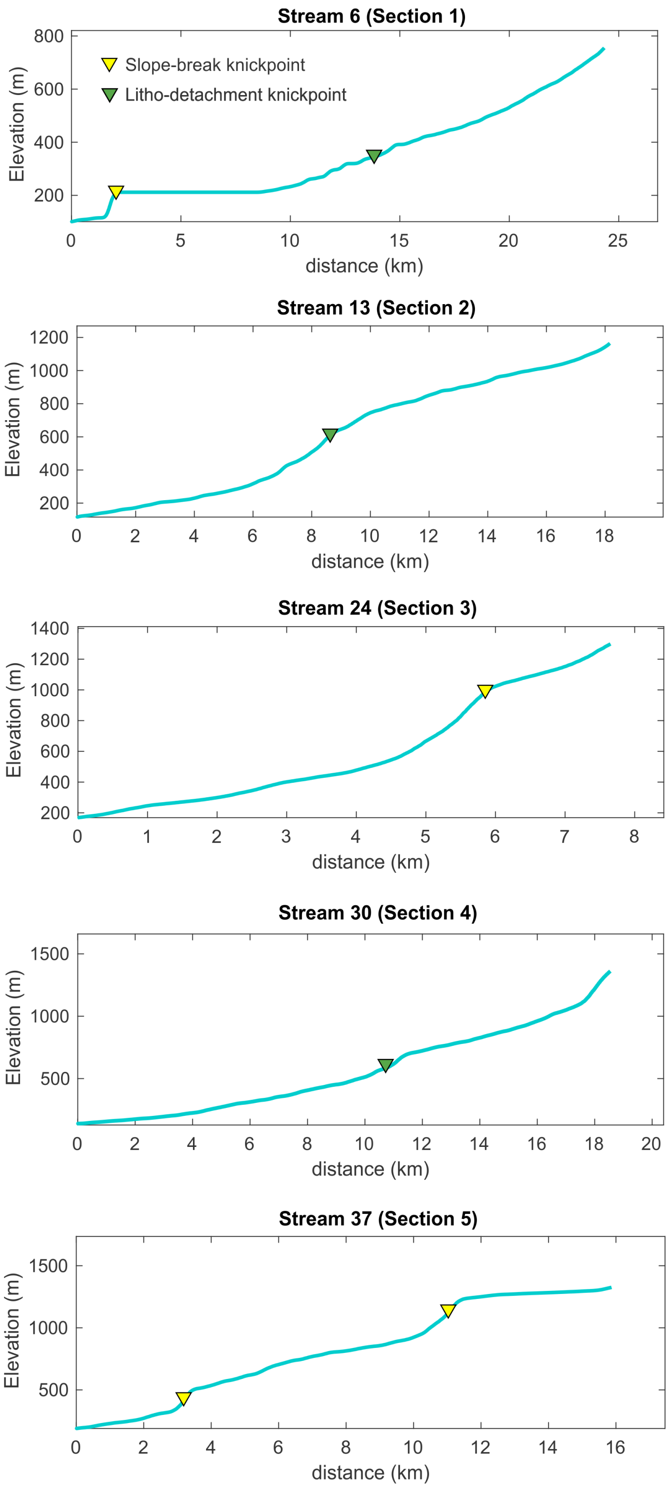

Figure S10. Examples of tectonic knickpoints (slope-break) and lithological knickpoints associated with the detachment faulting from each segment in the study area. All stream profiles are trunk rivers of basins.

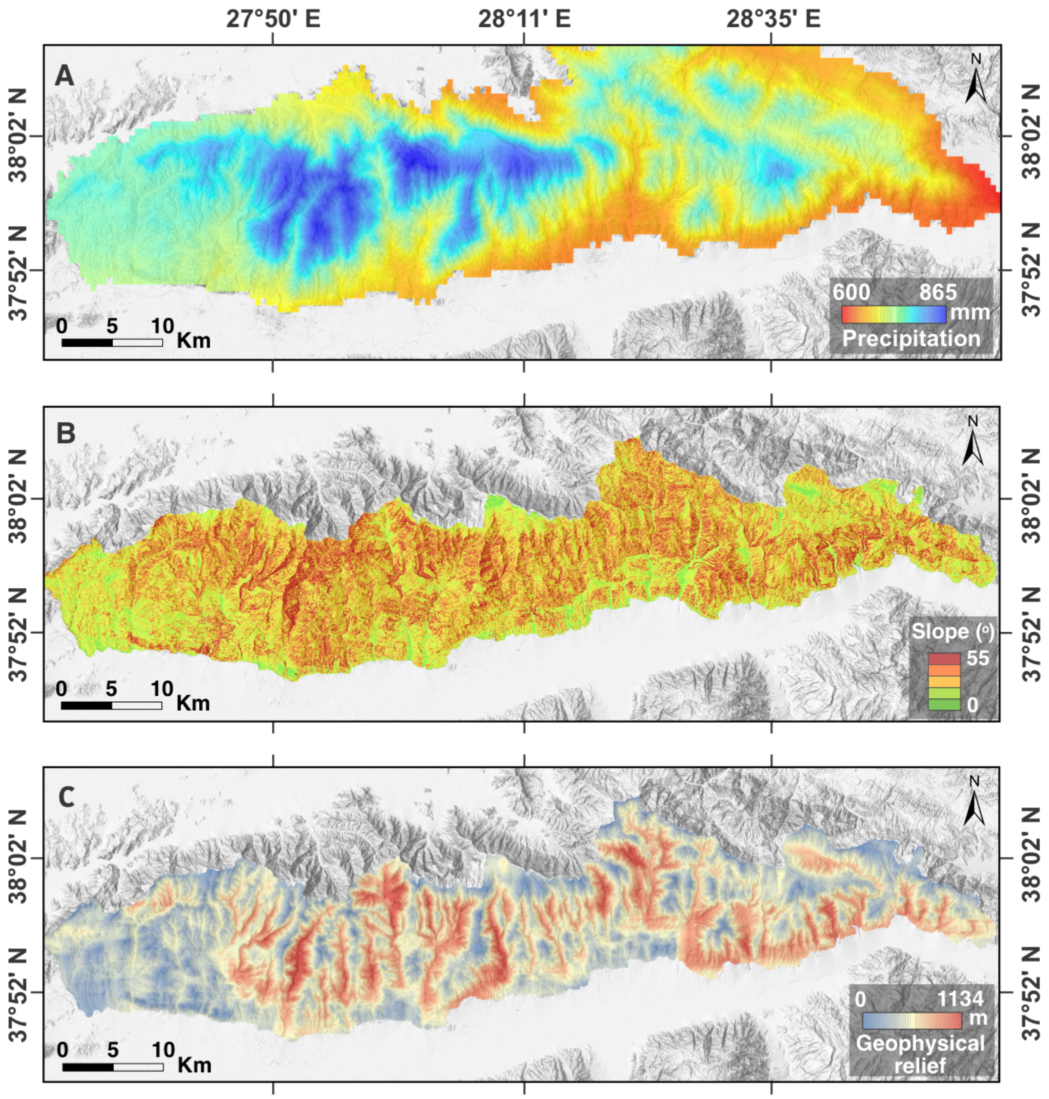

Figure S11. (A) Mean annual precipitation, (B) slope, and (C) geophysical relief of Aydın Range.

Table S1. Applied ED measurement protocols to samples. SAR sequence for quartz. In the case of observing any felspathic signal contribution, one more IR stimulation step at 50°C 100s is added between preheating and OSL stimulation steps.

Step	Treatment	Observed
**1**	Dose, D_i	
**2**	Preheat, 220°C, 5°C/s, 10 s	
**3**	OSL, 600s at 125°C, 5 °C/s	*Li*
**4**	Test dose, D_t	
**5**	Cut heat, 180°C, 5°C/s	
**6**	OSL, the 60s at 125°C, 5°C/s	*Ti*
**7**	Illumination, Blue 100s at 280 °C	
	Return 1st step	

Table S2. OSL dating results, sample locations, radioisotopes concentrations, cosmic, and total dose rates, DE estimates, and ages.

Terrace No	Sample ID	Latitude (°N)	Longitude (°E)	Elevation (m)	Depth (m)	U (ppm)	Th (ppm)	K (%)	Rb (ppm)	Aliquots	Dose rate (mGy/a)	Equivalent dose (Gy)	Over-dispersion (%)	Age (ka)
**Tabakhane River**														
**1**	TBK-E1	37.86068	27.84846	185	3.8	0.9	11.3	1.72	91.1	24	2.21± 0.11	153.21± 20.95	13±2	69 ± 10
**3**	TBK-E2	37.86555	27.84958	158	4.4	1.6	13.1	1.83	84.4	24	2.47± 0.13	130.11±25.23	19±3	47 ± 6
**Kabaklık River**														
**2**	KBK-W1	37.90140	27.98058	807	1.3	2.3	13.8	2.20	102.3	24	3.810± 0.264	101.28±11.14	11±2	34 ± 4
**3**	KBK-W2	37.89009	27.97616	185	2	2.0	12.3	2.38	104.4	24	3.699± 0.239	33.28± 5.21	15±2	11 ± 2
**Esnek River**														
**1**	ESN-W1	37.98861	28.68813	426	4.2	3.6	13.7	2.31	92.2	24	3.17± 0.16	121.34±15.8	28±4	38 ± 6
**1**	ESN-E2	37.99323	28.69408	401	6	2.6	10.3	1.57	62.1	24	2.22± 0.1	78.91±9.95	12±2	36 ± 5
**3**	ESN-W3	37.96880	28.68658	252	1.3	5.1	17.2	2.09	104.8	24	3.55± 0.16	22.23±5.88	26±4	6 ± 2
**4**	ESN-W4	37.98112	28.69045	380	4	6.4	26.3	2.66	153.5	24	4.62± 0.21	118.71±32.85	27±4	26± 7

Table S3. River incision rates, terrace height above the current river, and terrace abandoned ages.

Terrace number	Section 1 (Tabakhane River Terraces)			Section 2 (Kabaklık River Terraces)			Section 5 (Esnek River Terraces)		
	Terrace Height from the current river (m)	Abandoned age (ka)	Incision rate (mm/a)	Terrace Height from current river (m)	Abandoned age (ka)	Incision rate (mm/a)	Terrace Height from current river (m)	Abandoned age (ka)	Incision rate (mm/a)
T1	73	69±10	1.06 ± 0.16	50			65*	38 ± 6-36 ± 5	1.81± 0.26
T2	70			18	34 ± 4	0.53 ± 0.09	60		
T3	30	47 ± 6	0.64 ± 0.09	6	11± 2	0.55 ± 0.13	35	26 ± 7	1.35 ± 0.36
T4	12						10	6 ± 2	1.67 ± 0.36

*The height represents the strath elevation from the current river bed. The surface height of T1 is 72 m from the current river bed. The incision rate was calculated based on the strath surface for T1 in Esnek River.

Table S4. ^{10}Be concentrations and catchment-wide erosion rates in the southern flank of the Aydın Range, western Turkey.

Sample	Latitude (°N)	Longitude (°E)	Sample elevation (m)	Mean catchment elevation (m)	Mean hillslope angle (°)	Catchment area (km^2)	10Be concentration (atoms/g)	10Be concentration-error (atoms/g)	Catchment-wide erosion rates (mm/ka)	Internal uncertainty (1σ) (mm/ka)	External uncertainty (1σ) (mm/ka)	Erosion rate (g/cm2/a)
AYD-1	37.99751	28.69309	430	1020	20	95	23140	895	273	10.6	24	0.0615
AYD-2	38.05971	28.62069	876	1020	20	95	22967	990	275	11.9	24.8	0.0679
AYD-3	37.94229	28.54768	240	1079	22	34	15038	572	438	16.7	38.4	0.11
AYD-4	37.93804	28.49667	355	868	29	8	10810	566	530	27.6	50.2	0.132
AYD-5	37.99852	28.38209	235	705	24	148	24431	946	209	8.1	18.4	0.0522
AYD-6	37.95595	28.62232	380	1117	25	16	13153	678	512	26.4	48.4	0.126
AYD-7	37.92996	27.97746	425	701	22	31	25465	873	200	8.82	17.2	0.0499
AYD-9	37.93833	27.62483	236	541	22	89	19440	725	236	7.84	20.7	0.0591

Table S5. Metrics of alluvial fans and catchments in the study area.

No.	Fan area (km^2)	Fan toe elevation (m)	Fan volume (km^3)	Mean fan thickness (m)	Mean fan slope (◦)	Catchment area (km^2)	Mean catchment k_{sn} ($m^{0.9}$)	Mean catchment relief (m)	Catchment precipitation (mm/a)
1	20.2	54.3	0.71	34.6	3.6	29.1	38	423	735
2	36.39	53.8	0.78	21.4	1.7	89.7	48	518	747
3	3.51	86.3	0.04	12.4	3.7	4.3	58	453	731
4	21.03	56.3	0.05	22.5	1.9	42.2	52	457	736
5	15.5	60.2	0.29	19.1	2.7	33.8	37	520	738
6	24.4	58.5	0.45	18.4	2.2	166.7	38	798	763
7	5.5	94.2	0.15	28.3	3.1	5.1	64	542	711
8	16.7	63.3	0.56	33.8	2.5	18.5	38	1053	757
9	22.4	62.7	0.42	18.7	5.1	70.7	66	1008	780
10	4.1	70.1	0.09	21.6	4.4	12.5	65	866	731
11	1.8	65.2	0.04	23.8	3.1	3.1	79	1026	762
12	1.1	76.6	0.02	24.3	4.4	13.8	22	638	688
13	6.4	69.1	0.13	20.9	3.3	45.8	23	924	770
14	10.1	71.2	0.32	31.9	3.3	31.6	65	1002	742
15	18.2	65.4	0.45	24.5	2.1	112.1	18	932	749
16	11.4	72.2	0.27	24.1	2.6	107.7	65	938	747
17	1.01	138.2	0.03	49.4	4.5	3.1	40	841	680
18	1.03	131.1	0.04	51.1	4.2	1.8	68	892	705
19	2.06	128.7	0.08	42.7	3.3	5.1	78	960	754
20	1	153.1	0.03	54.6	5.8	1.3	86	978	725
21	1	154.8	0.03	48.8	5.7	1.6	76	988	695
22	1	154.5	0.08	76.1	6.2	2.8	77	1005	720
23	1.3	136.9	0.07	54.7	6.4	5.9	78	1022	742
24	2.5	130	0.27	79.4	6.8	6.3	79	1092	738
25	13.2	83.9	0.21	15.5	2.1	76.6	76	943	753

26	7.6	85.8	0.28	37.9	3.5	28.1	66	810	723
27	3.4	86.2	0.04	13.7	2.2	7.6	67	618	656
28	4.4	89.2	0.07	17.7	3.1	43.8	48	904	738
29	1.8	92.5	0.02	15.1	3.3	5.2	77	554	634
30	3.3	95.4	0.06	19.7	3.7	35.1	30	858	713
31	11.2	101.2	0.29	26.7	6.1	45.2	63	964	702
32	7.1	108.7	0.28	19.6	3.7	13.7	74	647	663
33	10.4	110.2	0.21	19.7	2.4	148.4	41	835	690
34	10.9	111.6	0.41	36.7	3.1	81.9	84	774	700
35	8.3	111.7	0.79	81.5	4.5	5.5	68	1056	695
36	8.4	116.9	0.45	54.8	2.9	57.9	73	840	709
37	5.9	123.4	0.17	29.8	2.9	34.3	62	953	725
38	3.9	124.3	0.13	34.4	3.2	8.8	78	1030	697
39	5.4	138.6	0.36	67.2	3.7	3.3	90	1036	669
40	7.8	142.7	0.71	90.7	3.8	16.1	69	1077	718
41	10.8	147.5	1.01	93.3	3.9	17.4	93	1123	706
42	8.2	160.9	0.44	54.1	3.5	97.9	82	778	703
43	9.1	169.3	0.74	81.1	4.6	34.6	63	733	692
44	5.5	167.9	0.31	55.7	3.2	7.7	77	1050	658
45	9.6	169.8	0.45	46.8	2.9	18.3	81	947	646
